# STAG2 Regulates Homologous Recombination Repair and Sensitivity to ATM Inhibition

**DOI:** 10.1002/advs.202302494

**Published:** 2023-11-20

**Authors:** Jie Zhou, Run‐Cong Nie, Zhang‐Ping He, Xiao‐Xia Cai, Jie‐Wei Chen, Wen‐ping Lin, Yi‐Xin Yin, Zhi‐Cheng Xiang, Tian‐Chen Zhu, Juan‐Juan Xie, You‐Cheng Zhang, Xin Wang, Peng Lin, Dan Xie, Alan D D'Andrea, Mu‐Yan Cai

**Affiliations:** ^1^ State Key Laboratory of Oncology in South China, Guangdong Provincial Clinical Research Center for Cancer Sun Yat‐sen University Cancer Center Guangzhou 510060 China; ^2^ Guangxi International Travel Healthcare Centre (Port Clinic of Nanning Customs District) Nanning Guangxi 530021 China; ^3^ Department of Gastric Surgery State Key Laboratory of Oncology in South China, Guangdong Provincial Clinical Research Center for Cancer Sun Yat‐sen University Cancer Center Guangzhou 510060 China; ^4^ Department of Pathology State Key Laboratory of Oncology in South China, Guangdong Provincial Clinical Research Center for Cancer Sun Yat‐sen University Cancer Center Guangzhou 510060 China; ^5^ Department of Thoracic Surgery State Key Laboratory of Oncology in South China, Guangdong Provincial Clinical Research Center for Cancer Sun Yat‐sen University Cancer Center Guangzhou 510060 China; ^6^ Department of Radiation Oncology Dana‐Farber Cancer Institute Boston MA 02215 USA; ^7^ Center for DNA Damage and Repair Dana‐Farber Cancer Institute Boston MA 02215 USA

**Keywords:** ATM inhibitors, homologous recombination, PARP inhibitors, STAG2, synthetic lethality

## Abstract

Stromal antigen 2 (STAG2), a subunit of the cohesin complex, is recurrently mutated in various tumors. However, the role of STAG2 in DNA repair and its therapeutic implications are largely unknown. Here it is reported that knockout of STAG2 results in increased double‐stranded breaks (DSBs) and chromosomal aberrations by reducing homologous recombination (HR) repair, and confers hypersensitivity to inhibitors of ataxia telangiectasia mutated (ATMi), Poly ADP Ribose Polymerase (PARPi), or the combination of both. Of note, the impaired HR by STAG2‐deficiency is mainly attributed to the restored expression of KMT5A, which in turn methylates H4K20 (H4K20me0) to H4K20me1 and thereby decreases the recruitment of BRCA1‐BARD1 to chromatin. Importantly, STAG2 expression correlates with poor prognosis of cancer patients. STAG2 is identified as an important regulator of HR and a potential therapeutic strategy for STAG2‐mutant tumors is elucidated.

## Graphical abstract

Ataxia telangiectasia mutated (ATM) inhibitors are potential antitumor drugs. Notably, the study illuminates that Stromal antigen 2 (STAG2) deficient cancer cells manifest heightened sensitivity to ATM inhibition. It also unveils a synthetic lethality existing between ATM and STAG2, due to increased DNA double‐strand breaks (DSBs). This culmination precipitates an inescapable DNA calamity, ultimately culminating in the demise of tumor cells.

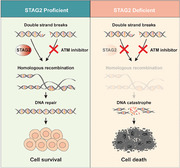

## 1 Introduction

The genomic DNA of eukaryotic cells is constantly challenged by endogenous or exogenous genotoxic sources, and cells have consequently evolved an intricately regulated DNA damage response (DDR) to safeguard against the damage.^[^ ^1^ ^]^ Among the various types of DNA lesions, DNA double‐strand breaks (DSBs) represent the most cytotoxic DNA lesions, which result from the cleavage of both strands of the double helix. In response to the DSBs, DDR is activated by ataxia telangiectasia mutated (ATM)‐dependent phosphorylation of cell cycle checkpoints.^[^ ^2^ ^]^ Depending on the cell‐cycle stage, ATM coordinates the activity of two DSB repair pathways: the homologous recombination (HR) pathway and the non‐homologous end joining (NHEJ) pathway. Since ATM plays such a key role in the DDR, the anti‐tumor effects of multiple ATM inhibitors (ATMis) have been widely investigated in both cancer cells and mouse models.^[^ ^3^ ^]^ Recently, we reported that knockout of both the Fanconi Anemia (FA)/BRCA pathway and ATM strongly inhibits end resection and generates toxic levels of NHEJ, thereby resulting in cellular death by synthetic lethality (SL). Intriguingly, single guide RNAs (sgRNAs) targeting Stromal Antigen 2 (STAG2) also conferred ATM inhibitor hypersensitivity.^[^ ^4^ ^]^

STAG2 is a member of the cohesin complex, composed of SMC1A, SMC3, and RAD21, that forms a ring‐shaped structure encircling the DNA double helix.^[^ ^5^ ^]^ The fourth core subunit of the complex is one of three members of the STAG family: STAG1, STAG2, or the meiosis‐specific paralogue STAG3.^[^ ^6^ ^]^ STAG2 and STAG1 are two mutually exclusive components of the cohesin complex that are essential for centromeric telomeric cohesion.^[^ ^7^ ^]^ The cohesin gene STAG2 plays important roles in a variety of processes beyond chromosome segregation, including chromatin organization, transcriptional regulation, and DNA replication and repair.^[^ ^8^ ^]^ Recurrent somatic mutations of cohesin genes have been identified across a wide spectrum of human tumors, including bladder cancer, endometrial cancer, glioblastoma, Ewing's sarcoma, and myeloid leukemia.^[^ ^9^ ^]^ Among the cohesin genes, STAG2 harbors the highest frequency of predicted pathogenic mutations in malignancies.^[^ ^10^ ^]^ STAG2 and TP53 mutations are found much more frequently in tumor cell lines derived from aggressive cancers, and patients with STAG2‐mutated Ewing's sarcoma have a higher rate of metastatic disease and worse outcomes.^[^ ^11^, ^12^ ^]^ Therefore, there is a great need for developing therapeutic strategies to improve the survival of tumor patients with STAG2 mutations.

Poly (ADP‐ribose) polymerase (PARP) inhibitors stand as the inaugural clinically endorsed pharmacotherapies strategically engineered to target malignancies characterized by BRCA1/2 deficiency, capitalizing on the principle of synthetic lethality. Presently, the clinical utility of PARP inhibitors has been extended to encompass a broader spectrum of cancers associated with HR deficiency, which encompasses genes such as PALB2, RAD51C, and others. However, it remains imperative to acknowledge that merely a minority fraction of cancer patients harbor HR deficiency, thereby constituting the subgroup that stands to gain therapeutic benefits from PARP inhibitor interventions.^[^ ^13^, ^14^, ^15^ ^]^ The therapeutic landscape of other DDR inhibitors has rapidly expanded to target the key sensors of DNA repair and replication, such as ATM, ATR, and WEE1. Bridging preclinical data on these agents to clinical use is crucial to inform predictive biomarker assays of responsiveness and assess the underlying mechanisms.^[^ ^1^ ^]^

Here, we demonstrate that STAG2 knockout in multiple tumor cell lines resulted in hypersensitivity to ATM inhibitors, which is consistent with our screening data. We further found that knockout of STAG2 led to increased DNA damage, impaired HR repair, enhanced chromosome aberrations, and decreased BRCA1 complex recruitment to chromatin. Since BRCA1 complexes recognize histone H4 tails specifically when they are unmethylated at lysine 20 (H4K20me0),^[^ ^16^, ^17^ ^]^ we reasoned that downregulation of STAG2 might influence the post‐translational methylation states of H4 lysine 20. Indeed, STAG2 knockout leads to reduced H4K20me0 protein levels by mediating methyltransferase KMT5A expression. More importantly, we found that tumor cells with STAG2‐knockout can be also selectively killed by an inhibitor of poly ADP ribose polymerase (PARPi), especially when combined with ATMi. Taken together, our study highlights an efficient HR repair regulatory mechanism of STAG2 and provides a promising therapeutic target for tumors with STAG2 mutations.

## 2 Results

### 2.1 STAG2 is Synthetically Lethal with ATM in Multiple Cancers

**Figure 1:**
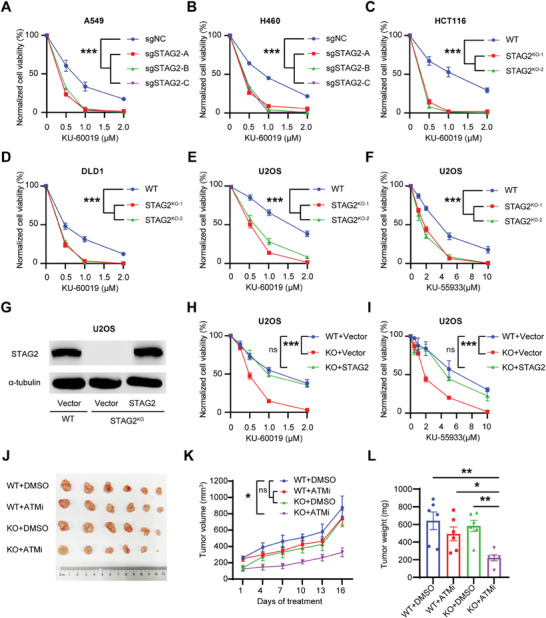
STAG2 is synthetically lethal with ATM inhibitors in multiple cancers. A,B) Clonogenic survival assays of control and knockout (KO) lung cancer cells after treatment with ATM inhibitor (KU‐60019) following transduction with STAG2‐targeting sgRNAs. ‐A, ‐B and ‐C indicate three independent sgRNAs. C–E) Clonogenic survival assays of colorectal cancer cells and U2OS cells after treatment with ATM inhibitor (KU‐60019) in stable clones of STAG2 knockout constructed via CRIPSR‐Cas9. F) Clonogenic survival assays of U2OS cells after treatment with the other ATM inhibitor (KU‐55933) in stable clones of STAG2 knockout via CRIPSR‐Cas9. G–I) Western blot and Clonogenic survival assays detecting the efficiency of STAG2 rescue and its impact after treatment with two ATMi (KU60019, KU55933) in U2OS cells. J–L) Tumor images, volumes and weight of xenograft tumors formed in nude mice (*n* = 6). Nude mice were injected subcutaneously with 2 × 10^6 DLD1 wildtype (WT) or STAG2 knockout (KO) cells, and intraperitoneally without or with ATM inhibitor (DMSO or 10 mg kg^−1^ KU‐55933) every three days for 16 days. Day 16 tumor images are shown. Dynamic tumor volumes were monitored and analyzed. Tumor weights of xenograft tumors formed in nude mice (n = 6). Data are shown as mean ± SEM. Statistical analysis was performed using one‐way and two‐way ANOVA (A, B, C, D, E, F, H, I, and K). ns, not significant, ^*^ *p*<0.05, ^***^ *p*<0.001.

Since STAG2 is emerging as one of 12 genes that are significantly mutated in human cancers,^[^ ^18^ ^]^ we first summarized the mutant frequency of STAG2 across multiple cancer types from cBioPortal (www.cbioportal.org). Consistent with the previous study, genomic analyses of the database demonstrated that STAG2 is most frequently mutated in bladder urothelial carcinoma (≈15%), followed by uterine corpus endometrial carcinoma (≈10%), stomach adenocarcinoma (≈5%), and lung adenocarcinoma (≈5%) (Figure S1A, Supporting Information).^[^ ^19^ ^]^ Recently, we demonstrated that transfection of single‐guide STAG2 RNAs (sgRNA) into lung cancer cells (i.e., A549 and H460) conferred ATM inhibitor sensitivity.^[^ ^4^ ^]^ To test whether STAG2 deficiency is synthetically lethal with ATM inhibitors, we validated the STAG2 sgRNA results and tested ATMi in multiple cell lines from STAG2‐mutant tumor types, including the identification of the original STAG2‐deficient cell line UMUC3 (Figure S1B, Supporting Information). As expected from the CRISPR screen, the depletion of STAG2 led to hypersensitivity to the ATMi, KU‐60019 (**Figure** **1**A–D; Figure S1C–F, Supporting Information). These findings were confirmed in the common model tumor lines U2OS and HeLa using two distinct ATMi drugs, KU‐60019 and KU‐55933 (Figure 1E,F; Figure S1G, Supporting Information). Next, we expressed STAG2 in STAG2‐knockout (STAG2^KO^) cells (Figure 1G; Figure S1H, Supporting Information), and observed that the restoration of STAG2 expression in STAG2^KO^ cells also restored resistance to ATMi (Figure 1H,I; Figure S1I, Supporting Information). Considering the role of STAG2 in the cohesin complex, we further investigated whether RAD21, another crucial protein within the complex, has a similar impact on ATMi sensitivity. Although RAD21 knockdown heightened the sensitivity to ATM inhibitor, the increased sensitivity was comparatively lower than that observed with STAG2 knockdown (Figure S1J, Supporting Information). This observation aligns with our CRISPR‐Cas9 screening data, indicating that the loss of STAG2 demonstrates a greater dependency on ATM compared to the depletion of RAD21 (dependency score: 9.56 vs 3.08).^[^ ^4^ ^]^ These findings might underscore the prominent role of STAG2 over other cohesin components in enhancing cell sensitivity to ATMi.

Moreover, in xenografts of DLD‐1 colorectal adenocarcinoma cells transduced with sgRNAs targeting STAG2 or control (non‐targeted sgRNA), knockout of STAG2 significantly inhibited tumor growth with ATMi treatment, as measured by tumor volume or tumor mass (Figure 1J–L). This synthetic lethal relationship shown in vitro and in vivo suggests that ATM inhibition is a potential therapeutic target for tumors with STAG2 defects.

### 2.2 STAG2 is Involved in DNA‐Double‐Stranded Break (DSB) Repair

**Figure 2:**
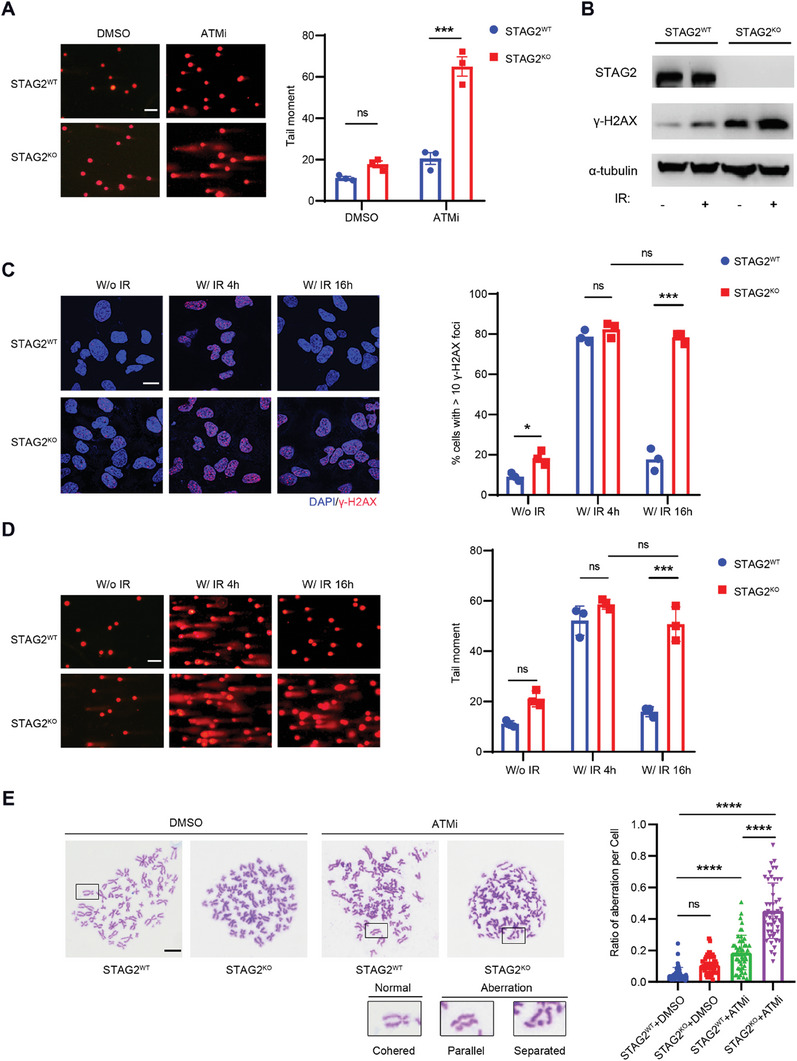
Knockout of STAG2 impairs DNA double‐strand breaks repair. A) Left: Representative images of neutral comets in STAG2^WT^ and STAG2^KO^ U2OS cells after treatment with DMSO or an ATM inhibitor (KU‐60019, 5 µM) for 24 h. Scale bar: 100 µm. Right: Quantification of the comet tail moment in left panel. At least 50 cells were counted in each condition. B) Western blot of the cell lysates from STAG2^WT^ and STAG2^KO^ U2OS cells 4 h after treatment without (W/o) or with (W/) irradiation (5 Gy). C) Left: Immunofluorescence images and quantification of *γ*‐H2AX foci in STAG2^WT^ and STAG2^KO^ U2OS cells at 4 and 16 h after 5 Gy irradiation treatment. Scale bar: 20 µm. Right: Percentage of the cells with >10 *γ*‐H2AX foci. At least 50 cells were counted in each condition. D) Left: Representative images of neutral comets in STAG2^WT^ and STAG2^KO^ U2OS cells at 4 and 16 h after 5 Gy irradiation treatment. Scale bar: 100 µm. Right: Quantification of the comet tail moments shown in the left panel. At least 50 cells were counted in each condition. E) Left: Representative images of chromosomal aberrations in metaphase spreads of STAG2^WT^ or STAG2^KO^ U2OS cells 4 h after treatment with 100 ng ml^−1^ colchicine following 24 h exposure to DMSO or ATM inhibitor (KU‐60019, 5 µM). Scale bar: 10 µm. Right: Quantification of chromosomal aberrations. At least 30 cells were counted in each condition. Data are shown as mean ± SEM from three independent experiments and were analyzed by one‐way ANOVA test (A, B, D, and E). ns, not significant, ^*^ *p*<0.05, ^***^ *p* <0.001, ^****^ *p* <0.0001.

An early response to DNA damage involves the activation of ATM, an essential kinase that initiates DSB repair in response to various DNA lesions.^[^ ^1^ ^]^ To examine whether STAG2 is involved in DSB repair, we exposed U2OS cells to ATM inhibition and analyzed the DNA breaks with a comet assay. Knockout of STAG2 resulted in increased DNA DSBs, as measured by ATMi‐induced longer comet tails (**Figure** **2**A). The results from the comet assay effectively demonstrate the synthetic lethality between STAG2 defects and ATMi, primarily linked to the accumulation of significant DNA double‐strand breaks. The UMUC3 cell line, which inherently exhibits STAG2‐deficient mutations, likewise exhibited a congruous pattern of response to ATMi treatment akin to that observed in STAG2^−/−^ U2OS cells (Figure S2A,B, Supporting Information). This substantiation assumes heightened significance due to the prevalent deletions characterizing STAG2 mutations, consequently bolstering the practical implications of our research findings. To determine whether the induction of DSB extends beyond ATMi to other DNA‐damaging agents, we examined the DNA DSB in response to irradiation (IR), camptothecin (CPT), and cisplatin. The deletion of STAG2 in either U2OS cells or HeLa cells conferred the sensitivity to all of the tested DNA‐damaging agents (Figure S2C–H, Supporting Information). DSBs in chromatin promptly initiate the phosphorylation of the histone H2A variant, H2AX, at Serine 139 to generate gamma‐H2AX (*γ*‐H2AX).^[^ ^20^ ^]^ The level of *γ*‐H2AX in STAG2^KO^ cells was higher after IR than in wild‐type (STAG2^WT^) cells (Figure 2B). Although STAG2 knockout did not alter the induction of *γ*‐H2AX foci, the percentage of *γ*‐H2AX‐foci‐positive cells in STAG2^KO^ cells was significantly higher than that of STAG2^WT^ cells 16 h after IR treatment (Figure 2C), suggesting increased DNA DSBs in STAG2‐deficient cells. Additionally, the tail moments of STAG2^KO^ cells were significantly longer than those of STAG2^WT^ cells at 16 h after IR treatment (Figure 2D). These findings suggest that loss of STAG2 indicates increased DSBs in tumor cells.

As an excess of DSBs poses a serious threat to genome integrity,^[^ ^21^ ^]^ we hypothesized that the DSBs caused by STAG2 knockout may lead to chromosomal aberrations or genome instability. We performed a metaphase chromosome spread experiment to visualize chromosomal abnormalities in wild‐type and STAG2^KO^ U2OS cells after treatment with or without ATMi. In the presence of ATMi, STAG2^KO^ cells exhibited increased chromosome abnormalities (Figure 2E). Taken together, these results imply that STAG2 plays a critical role in DNA repair, the deficiency of which increases the accumulation of DSBs and genome instability, and finally leads to the synthetic lethality with ATM.

### 2.3 STAG2 Facilitates Homologous Recombination Repair

In light of our observations that STAG2 plays a crucial role in genome instability, we considered the possibility that STAG2 may play an important role in DNA repair. The existing literature has previously elucidated the involvement of STAG2 in DNA repair processes, primarily within the framework of its role in replication fork progression.^[^ ^22^ ^]^ In our endeavor to discern the precise functional landscape of STAG2 within the realms of both replication fork progression and DSB repair, we embarked upon a comprehensive comparative analysis. This involved meticulous scrutiny of the modulation of key kinases and proteins that distinctly govern the DSB damage response and replication fork progression. Our meticulous evaluation unveiled a compelling pattern, wherein STAG2's pronounced regulatory impact is notably concentrated within the context of DSB repair. Intriguingly, our findings reveal an augmented phosphorylation status of ATM, CHK2, and p53 proteins, which bear pivotal roles in DSB repair pathways. In contrast, the phosphorylation levels of ATR and CHK1, pivotal in the context of replication fork collapse, exhibited minimal changes within STAG2‐deficient U2OS and HeLa cells (Figure S3A–K, Supporting Information). This distinctive profile engenders a pronounced inclination toward investigating STAG2's role in DSBs, reinforcing its salience in this specific context.

**Figure 3:**
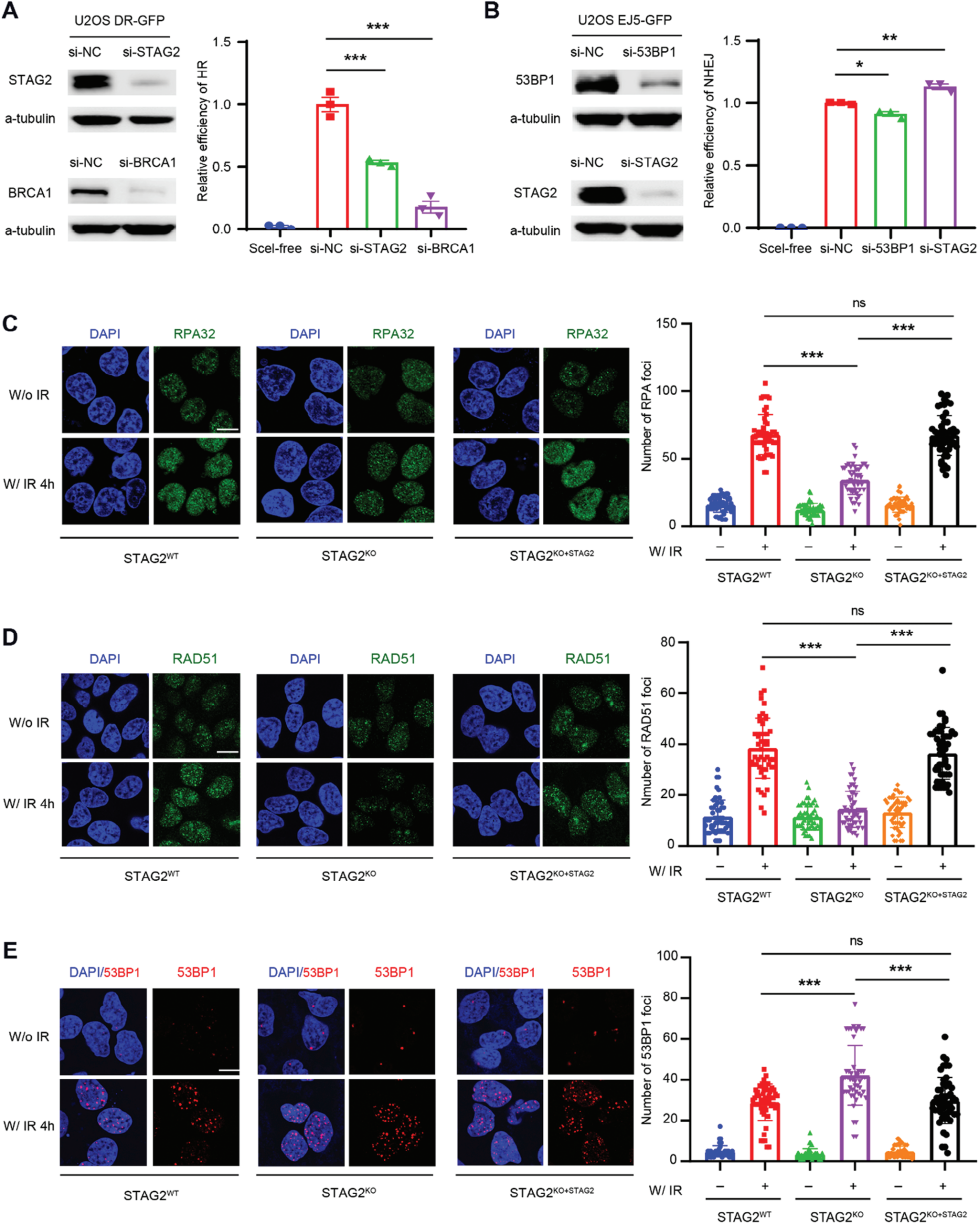
Knockout of STAG2 decreases homologous recombination repair. A) Left: Western blot of STAG2 and BRCA1 expression in the U2OS‐DR‐GFP cells after the treatment with si‐Control (NC), si‐BRCA1, and si‐STAG2. Right: The homologous recombination efficiency was measured using the U2OS‐DR‐GFP cell line. Cells were transfected with si‐NC, si‐STAG2, or si‐BRCA1, exposed to I‐Scel adenovirus or not (Scel‐free) for 6 h, and cultured with fresh medium. GFP‐positive cells were quantified after 72 h using FACS. B) Left: Western blot of STAG2 and 53BP1 expression in the U2OS‐EJ5‐GFP cells after the treatment with si‐NC, si‐53BP1, and si‐STAG2. Right: The non‐homologous end joining efficiency was measured using the U2OS‐EJ5‐GFP cell line. Cells were transfected with si‐NC, si‐53BP1, or si‐STAG2, exposed to I‐Scel adenovirus or not (Scel‐free) for 6 h, and cultured with fresh medium. GFP‐positive cells were quantified after 72 h using FACS. C–E) Immunofluorescence images and quantification of RPA32, RAD51, and 53BP1 foci 4 h after treatment without (W/o) or with irradiation (W/IR, 5 Gy) in STAG2^WT^, STAG2^KO^, and STAG2^KO+STAG2^ U2OS cells. At least 50 nuclei were counted in each condition. Data are shown as mean ± SEM and were analyzed by one‐way ANOVA. ns, not significant, ^*^ *p* <0.05, ^**^ *p* <0.01, ^***^p <0.001.

The analysis of subcellular fractions indicated that STAG2 levels, as well as *γ*‐H2AX and other proteins involved in DNA repair, persisted longer in chromatin fractions after irradiation (Figure S3L, Supporting Information). DSBs are the most genotoxic type of DNA damage and can be mainly repaired by error‐prone NHEJ repair pathways and error‐free HR pathways.^[^ ^23^, ^24^ ^]^ To determine the effect of STAG2 on DSB repair, we utilized the direct‐repeat‐green fluorescent protein (DR‐GFP) and end‐joining 5‐GFP (EJ5‐GFP) reporter assays to quantify the activities of HR and NHEJ, respectively, in U2OS cells. The reporter assays allow us to measure repair via HR or NHEJ of a DSB produced by the endonuclease I‐SceI in GFP‐positive cells.^[^ ^25^, ^26^ ^]^ Knockdown of STAG2 in U2OS cells resulted in a decrease in HR activity and a slight increase in NHEJ activity when compared to the control cells (**Figure** **3**A,B). Moreover, si‐RAD21 indeed impeded HR but less than si‐STAG2, and its impact was markedly less potent when contrasted with the profound influence exerted by si‐STAG2 (Figure S3M, Supporting Information). Similarly, combining shRNA targeting STAG2 with si‐RAD21 resulted in a further reduction in HR efficiency (Figure S3N, Supporting Information). These findings together underscore the exceptional role of STAG2 in the context of HR, surpassing other cohesin proteins, and positioning it as a linchpin in the preservation of genomic stability.

To further examine whether the loss of STAG2 caused a defect in HR repair, we investigated the accumulation of HR core factors RPA32 and RAD51, which play critical roles in HR repair through DNA end‐resection and filament formation.^[^ ^27^ ^]^ STAG2^−/−^ cells exhibited a significant decrease in RPA32 and RAD51 foci formation after induction of DBS by irradiation. Conversely, the re‐expression of STAG2 in STAG2^−/−^ cells increased the RPA32 and RAD51 foci formation (Figure 3C,D). Besides, a natural STAG2 deficient cell, UMUC3, also showed an increase of RPA32 and RAD51 foci after stably expressing STAG2 protein (Figure S3O,P, Supporting Information), these findings together exhibited an importance of STAG2 in maintaining an unobstructed HR process.

Since 53BP1 is another key DSB‐responsive protein promoting the repair of DSB by NHEJ while preventing HR^[^ ^28^ ^]^ we examined the formation foci of 53BP1 following exposure to IR. As expected, STAG2^−/−^ U2OS cells showed an increase in 53BP1 foci formation, and the rescue of STAG2 expression resulted in a reduction in 53BP1 foci (Figure 3E). Cohesin including STAG2 is important for transcriptional regression in response to DNA DSBs.^[^ ^29^ ^]^ We next investigated whether STAG2 deficiency influenced the expression levels of core factors in DSB repair. STAG2 knockout increased remarkable *γ*‐H2AX expression (i.e., DSB damage), but had no apparent impact on the expression levels of HR key proteins such as RPA32 and RAD51 (Figure S3Q, Supporting Information). Taken together, our findings confirm an important functional role of STAG2 in mediating DNA HR repair by accumulating HR‐related factors to DSB sites.

### 2.4 STAG2 Promotes the Recruitment of Brca1 to Chromatin via Regulating Kmt5a Expression and Downstream H4k20 Methylation

**Figure 4:**
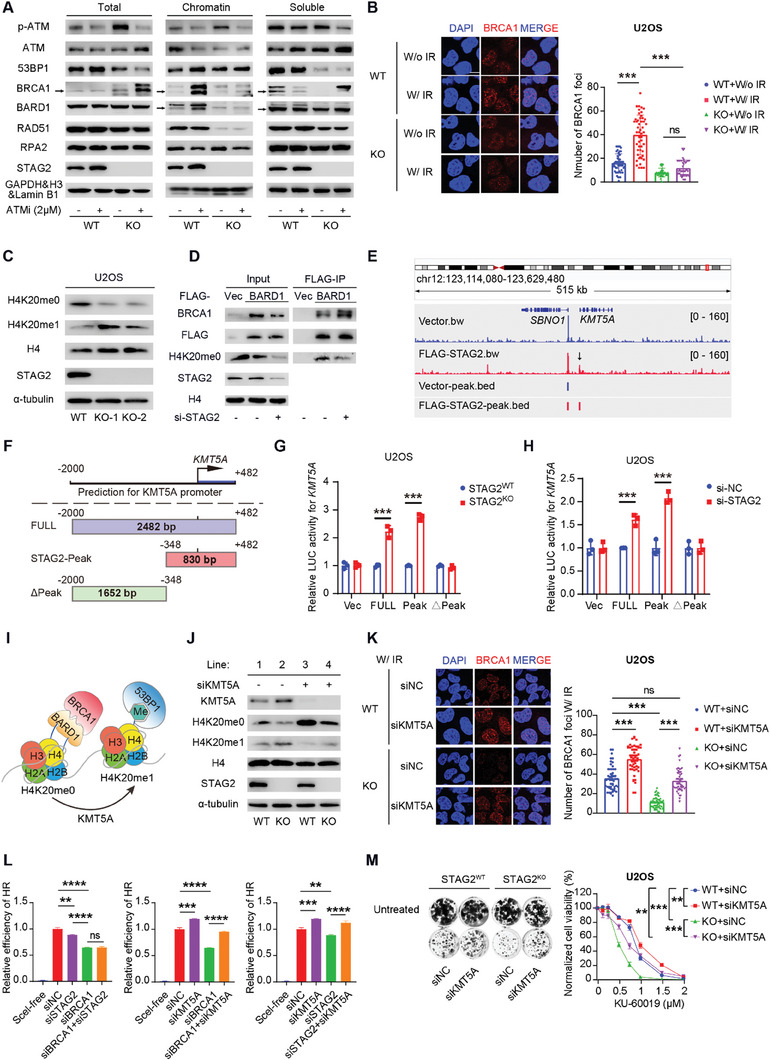
STAG2 influences the recruitment of BRCA1 and BARD1 to DNA damage site by regulating H4K20 methylation mediated by KMT5A. A) Western blot of the indicated proteins in subcellular fractions of STAG2^WT^ and STAG2^KO^ U2OS cells after treatment with 2 mM ATMi (KU‐60019) for 24 h. GAPDH was used as the control for the total cell lysates. Histone H3 was used as the control for the nucleus chromatin proteins. Lamin B1 was used as the control for the nucleus‐soluble proteins. Arrows indicate the target bands. B) Left: Immunofluorescence images of BRCA1 foci 4 h after treatment without (W/o) or with irradiation (W/IR, 5 Gy) in STAG2^WT^ and STAG2^KO^ U2OS cells. Right: Quantification analysis of BRCA1 foci 4 h after treatment without (W/o) or with irradiation (W/IR, 5 Gy) in STAG2^WT^ and STAG2^KO^ U2OS cells, related to left panel. At least 50 nuclei were randomly counted in each group. C) Western blot of the indicated proteins from STAG2^WT^ and STAG2^KO^ U2OS cell lines. D) Western blot showing FLAG‐immunoprecipitation of FLAG‐BARD1 in U2OS cells treated with siRNA negative control and si‐STAG2. E) CUT & Tag‐seq for FLAG or FLAG‐STAG2 at the KMT5A gene locus (indicated by the black arrow) in U2OS cells. The bed files showed the detected signal of vector or FLAG‐STAG2 peaks. F) A schematic diagram of the luciferase reporter plasmids constructed with three different fragments of the KMT5A promoter. STAG2‐Peak indicates the specific sequence of STAG2 binding to KMT5A which is obtained by CUT‐Tag sequencing. G) Relative values of luciferase (LUC) activity measured after transfection of different reporter plasmids in wild‐type (STAG2^WT^) or STAG2 knockout (STAG2^KO^) U2OS cells (Relative to STAG2^WT^ each group). H) Relative values of luciferase (LUC) activity measured after transfection of different reporter plasmids in U2OS cells followed by si‐NC or si‐STAG2 (Relative to si‐NC each group). I) A cartoon showing that BRCA1‐BARD1 complex and 53BP1 interact with different methylated H4 histones. J) Western blot of the indicated proteins from STAG2^WT^ and STAG2^KO^ U2OS cells treated with siRNA negative control and si‐KMT5A. K) Left: Immunofluorescence images and quantification of BRCA1 foci 4 h after treatment with irradiation (W/IR, 5 Gy) in STAG2^WT^ and STAG2^KO^ U2OS cells treated with siRNA negative control and si‐KMT5A. Right: Quantification of BRCA1 foci 4 h after treatment with irradiation (W/IR, 5 Gy) in STAG2^WT^ and STAG2^KO^ U2OS cells with siRNA negative control and si‐KMT5A, related to the left panel. At least 50 nuclei were randomly counted in each group. L) Quantification of HR efficiency using direct repeat U2OS DR‐GFP reporter assay. Cells were treated with si‐NC, si‐STAG2, si‐BRCA1, si‐KMT5A, si‐BRCA1+si‐STAG2, si‐BRCA1+si‐KMT5A, and si‐STAG2+si‐KMT5A. M) Left: Representative images of colony formation by STAG2^WT^ or STAG2^KO^ U2OS cells treated with si‐NC and si‐KMT5A, and treated with DMSO or ATMi (KU‐60019, 1 µM). Right: Clonogenic survival assays of STAG2^WT^ and STAG2^KO^ U2OS cells treated with siRNA negative control and si‐KMT5A and treated with DMSO or ATMi (KU‐60019). Data are shown as mean ± SEM from three independent experiments and were analyzed using the one‐way ANOVA test (B, G, H, K, and L), and two‐way ANOVA (M). ns, not significant, *p<0.05, **p<0.01, ***p < 0.001.

To explore the underlying mechanism of STAG2 in promoting DSB HR repair, we examined the chromatin recruitment of key DSB repair‐related proteins (including ATM, 53BP1, BRCA1, BARD1, RAD51, and RPA2) after depletion of STAG2 and/or ATMi treatment. When STAG2 was knocked out, the expression level of phosphorylated‐ATM (p‐ATM) was increased both in total lysates or chromatin accumulation, and the loss of STAG2 remarkably decreased the chromatin‐accumulated RAD51 (**Figure** **4**A). Interestingly, decreased chromatin accumulations of BRCA1 and BARD1 were observed in STAG2‐depleted cells regardless of ATMi treatment (Figure 4A). In support of the chromatin‐accumulated fractions, STAG2^−/−^ cells had a defect in BRCA1 foci assembly after induction by IR (Figure 4B). Together, these results suggest that STAG2 promotes HR repair by mediating chromatin accumulations of BRCA1 and BARD1.

The post‐replication chromatin of methylation switch at histone 4 lysine 20 (H4K20) governs the attachment of reader engagements (BRCA1 or 53BP1) to competing DSB repair pathways, HR or NHEJ. BRCA1–BARD1 complex recruitment to chromatin requires its binding to histone H4 unmethylated at lysine 20 (H4K20me0), thereby promoting HR.^[^ ^16^, ^17^ ^]^ We hypothesized that STAG2 might regulate BRCA1/BARD1 recruitment to chromatin by regulating levels of H4K20me0. As anticipated, the depletion of STAG2 led to a reduction in H4K20me0 and an increase in H4K20me1, observed both in the knockout and naturally STAG2‐deficient cell lines (Figure 4C; Figure S4A, Supporting Information). Moreover, STAG2^−/−^ cells exhibited an augmentation in the BARD1‐BRCA1 interaction, accompanied by a decrease in the BARD1‐H4K20me0 interaction (Figure 4D).

To explore how STAG2 mediates BRCA1‐BARD1 recruitment to chromatin, we performed a gene set enrichment analysis (GSEA) between STAG2‐positive and STAG2‐negative groups from the TCGA database. GSEA revealed that the chromatin‐modifying enzyme pathway was highly enriched in the STAG2‐positive population, indicating that STAG2 expression may influence chromatin modifications (Figure S4B,C, Supporting Information). We next selected six major methyl‐modified enzymes including three methyl‐writers (KMT5A, KMT5B, and KMT5C^[^ ^30^ ^]^) and three methyl‐erasers (PHF8,^[^ ^31^ ^]^ KDM1A,^[^ ^32^ ^]^ and KDM4A^[^ ^33^ ^]^), which have the potential for switching the methylation status of H4K20, and evaluated their mRNA expression after STAG2 knockout. Among them, KMT5A (Lysine Methyltransferase 5A) exhibited the largest increase in expression level after the depletion of STAG2 in U2OS or HeLa cells (Figure S4D–F, Supporting Information). Furthermore, our CUT & Tag sequencing data notably unveil the strategic association of STAG2 peaks with the KMT5A locus, thus providing insights into the precise region of STAG2‐specific binding (Figure 4E). This informative foundation facilitated the generation of three distinct plasmids, each harboring the full‐length predicted promoter of KMT5A, the STAG2‐specific binding sequence, and the residual sequence, respectively (Figure 4F). Notably, luciferase reporter assays conducted subsequent to STAG2 expression knockdown or knockout revealed a heightened luciferase activity exhibited by both the full‐length promoter and the STAG2‐specific binding sequence (Figure 4G,H; Figure S4G,H, Supporting Information). This discernible enhancement in luciferase activity serves to corroborate and validate the outcomes derived from our sequencing analyses, thereby substantiating the direct influence wielded by the STAG2 protein upon the cis‐elements governing KMT5A.

KMT5A is a writer of histone methylation and can convert unmethylated H4K20 into mono‐methylated H4K20 (H4K20me1) (Figure 4I).^[^ ^34^ ^]^ Therefore, STAG2 expression may facilitate HR by downregulating KMT5A‐mediated H4K20 methylation. Accordingly, loss of STAG2 would result in increased KMT5A‐mediated methylation of H4K20 and decreased HR activity. As predicted, the knockdown of KMT5A resulted in a significant increase in H4K20me0 and a decrease in H4K20me1 either in STAG2^WT^ cells or in STAG2^KO^ cells (Figure 4J). More importantly, the knockdown of KMT5A in STAG2^KO^ cells exhibited an increase in BRCA1 foci formation (Figure 4K). These data suggest that STAG2 can promote BRCA1 recruitment to chromatin via regulating KMT5A for the H4K20 methylation switch.

To assess further the effect of KMT5A on HR in the context of STAG2, we utilized the DR‐GFP reporter assay to examine HR activity after KTM5A silencing in STAG2 knockdown cells. Knockdown of STAG2 did not reduce further the efficiency of HR in BRCA1‐deficient cells (Figure 4L), indicating that the function of STAG2 in HR may depend on BRCA1. Importantly, the depletion of KMT5A enhanced the activity of HR, in the context of both STAG2 knockdown and BRCA1 knockdown (Figure 4L). The knockdown of KMT5A increased the resistance of STAG2^KO^ cells to ATMi, as shown by colony formation (Figure 4M; Figure S4I, Supporting Information). Taken together, these data suggest that STAG2 can inhibit the expression of KMT5A and maintain the unmethylation status of H4K20, which is required for BRCA1‐BARD1 recruitment to the DNA damage site for HR repair.

### 2.5 ATM Inhibition Sensitizes STAG2‐Depleted Tumors to the Treatment of PARP Inhibition

**Figure 5:**
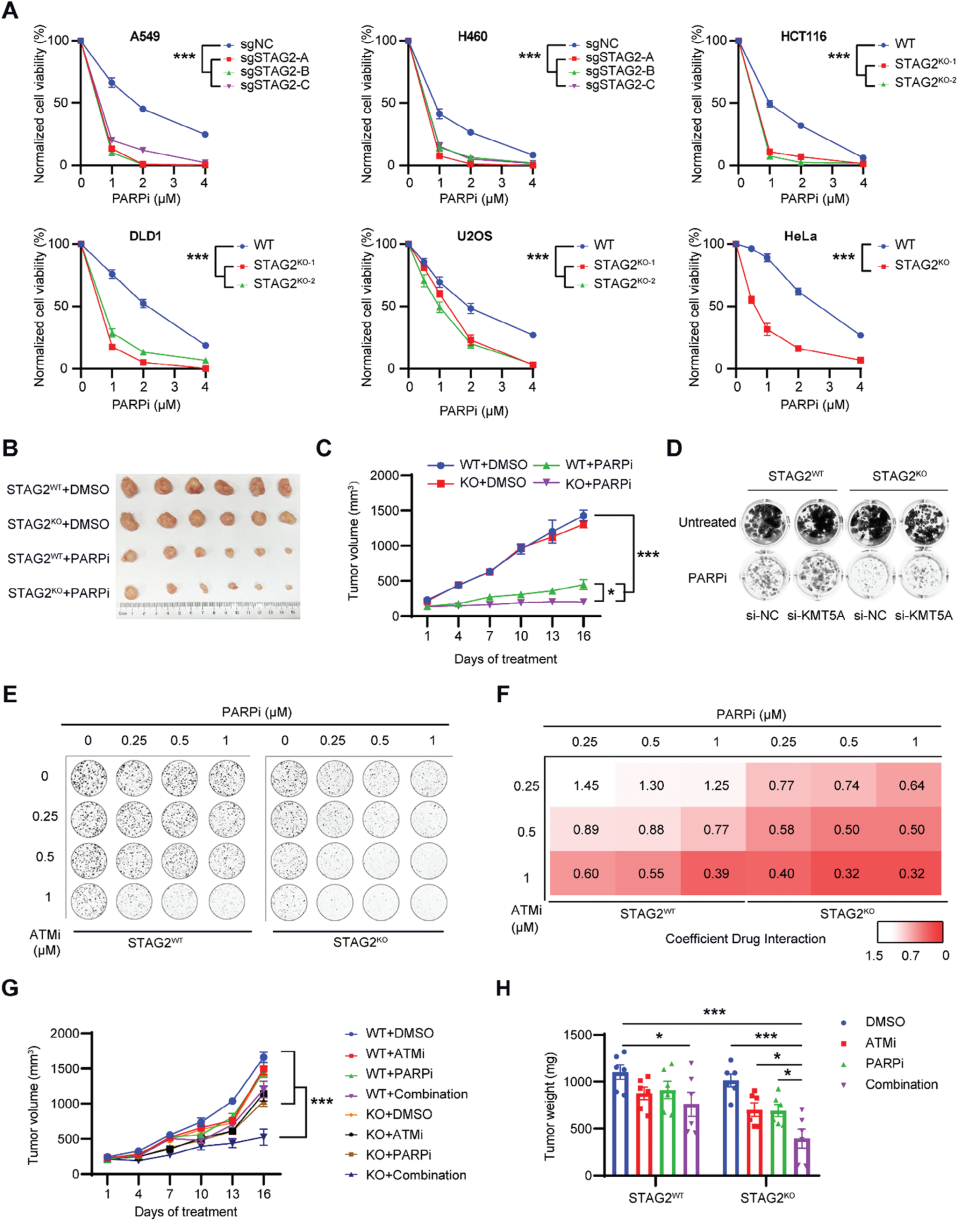
Knockout of STAG2 increases the sensitivity of PARPi and improves the synergy effect of ATMi and PARPi combination in vitro and in vivo. A) Clonogenic survival assays of multiple cancer cells treated with PARP inhibitor (PARPi, Olaparib) after sg‐STAG2, stable clones of STAG2 knockout via CRIPSR‐Cas9. B,C) Tumor images and volumes of xenograft tumors formed in nude mice (*n* = 6). Nude mice were injected subcutaneously with 2 × 10^6 DLD1 STAG2^WT^ or STAG2^KO^ cells, and intraperitoneally with or without PARP inhibitor (DMSO or 50 mg kg^−1^ AZD2281) every three days for 16 days. An image of day 16 tumors is shown. Dynamic tumor volumes were monitored and analyzed. D) Representative images of colony formation by STAG2^WT^ or STAG2^KO^ U2OS cells treated with si‐NC and si‐KMT5A and treated with DMSO or PARPi (Olaparib, 2 µM). E,F) Representative images of colony formation by STAG2^WT^ or STAG2^KO^ DLD1 cells after treatment with gradient concentrations of ATMi (KU‐60019) and PARPi (Olaparib). Coefficient of drug interaction of combined treatment with ATMi and PARPi in STAG2^WT^ or STAG2^KO^ DLD1 cells. Coefficient of drug interaction (CDI) < 1 indicates synergistic effect, CDI < 0.7 indicates good synergistic effect, CDI = 1 indicates no synergy, and CDI > 1 indicates antagonism. G,H) Tumor volumes and weights of xenograft tumors in nude mice (*n* = 6). Nude mice were injected subcutaneously with 2 × 10^6 DLD1 STAG2^WT^ or STAG2^KO^ cells intraperitoneally with DMSO, ATMi (KU‐55933, 5 mg kg^−1^), PARPi (Olaparib, 25 mg kg^−1^), or a combination of ATMi and PARPi (5 mg kg^−1^ KU‐55933 + 25 mg kg^−1^ Olaparib) three days for 16 days. Dynamic tumor volumes were monitored and analyzed. Day 16 weights are shown. Data are shown as mean ± SEM, and were analyzed by two‐way ANOVA test (A, C, and G), and one‐way ANOVA test (H). ^*^ *p*<0.05, ^***^ *p*<0.001.

Tumor cells with BRCA1/2 mutations or HR defects are sensitive to PARPis,^[^ ^35^, ^36^, ^37^, ^38^ ^]^ and recent studies indicated that STAG2 status correlates with treatment response to PARP inhibitors.^[^ ^39^, ^40^ ^]^ Since knockout of STAG2 impairs HR activity, we determined whether STAG2 knockout enhances the sensitivity to PARPi. Consistently, depletion of STAG2 with siRNA or sgRNA increased the sensitivity to the PARPi Olaparib in a panel of STAG2^KO^ and STAG2^WT^ cell lines (**Figure** **5**A; Figure S5A, Supporting Information). Conversely, overexpression of STAG2 in the cancer cell line with an inactivating mutation of STAG2 (i.e., UMUC3) resulted in PARPi resistance (Figure S5A, Supporting Information). Moreover, the DNA alkaline comet assay showed that Olaparib induced an increase in DSBs in STAG2^KO^ cells (Figure S5B, Supporting Information). Our findings suggest that STAG2 deficiency acts as a synthetic lethal with PARPi and causes more DSBs induced by PARP inhibition. Furthermore, knockout of STAG2 inhibited tumor growth in vivo after the treatment with PARPi (Figure 5B,C; Figure S5C,D, Supporting Information). Knockdown of KMT5A induced the resistance to Olaparib in STAG2^KO^ cells (Figure 5D; Figure S5E, Supporting Information), further indicating the important role of KMT5A in STAG2‐mediated DSB repair.

Given that the knockout of STAG2 sensitized tumor cells to ATMi and PARPi, we hypothesized that ATM inhibition may synergize in vitro and in vivo with PARP inhibition to promote tumor cell killing. To avoid overshadowing the combined effects of the two drugs with a dominant single‐drug dosage, we deliberately halved the concentrations of both drugs, reducing them to half of the single‐drug dosage. We observed a decreased inhibitory effect on tumor tissue with the reduced ATMi dosage, albeit less pronounced than that of the higher dosage. However, in the presence of PARPi, the STAG2^KO^ DLD1 cells exhibited heightened sensitivity to ATM inhibition, suggesting a synergistic effect with a combination index of 0.32 (Figure 5E,F, Supporting Information). Of note, the combined treatment showed minimal effect on STAG2^WT^ cells (Figure 5E,F). Moreover, the combination treatment of ATMi and PARPi led to significant suppression of tumor growth of the STAG2^KO^ tumors in vivo, evaluated either by tumor volume or by tumor weight (Figure 5G,H; Figure S5F,G, Supporting Information); however, there was a limited synergistic effect of the combined treatment on the growth of the STAG2^WT^ tumors. Together, our results suggest that PARP inhibition further sensitizes the STAG2‐deficient tumors to ATM inhibition.

### 2.6 STAG2 Functionally Contributes to Tumor DNA Damage Repair and Correlates with Tumor Patient Prognosis

**Figure 6:**
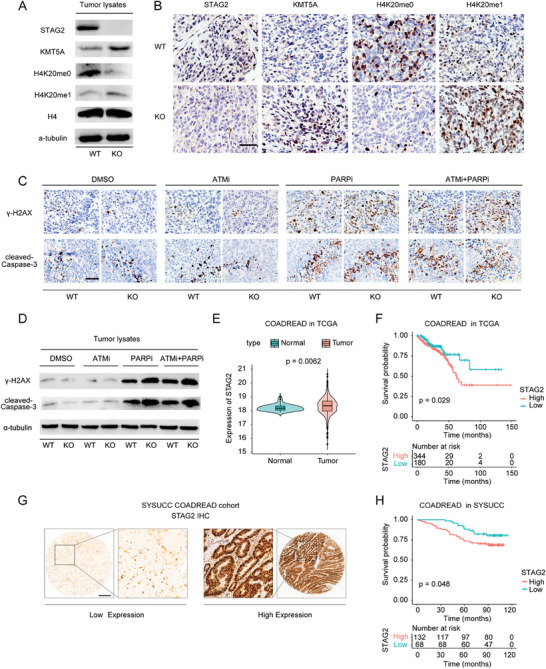
STAG2‐deficient xenograft tumors exhibit higher DNA damage after treatment with ATMi and PARPi, and high STAG2 indicates a poor clinical outcome. A) Western blot of the indicated proteins from xenograft tumor lysates. Tissues were derived from xenograft tumors that were subcutaneously treated with STAG2^WT^ and STAG2^KO^ DLD1 cells. B) Representative immunohistochemical images of STAG2, KMT5A, H4K20me0, and H4K20me1 in fixed tumor tissues from xenograft tumors. Tissues were derived from xenograft tumors that were subcutaneously treated with STAG2^WT^ and STAG2^KO^ DLD1 cells. Scale bars: 100 µm. C,D) Representative immunohistochemical images and western blot of *γ*‐H2AX and cleaved Caspase‐3 in fixed tumor tissues and lysates from xenograft tumors, respectively. Tissues were derived from xenograft tumors that were subcutaneously treated with STAG2^WT^ and STAG2^KO^ DLD1 cells following the treatment with DMSO, ATMi (KU‐55933, 5 mg kg^−1^), PARPi (Olaparib, 25 mg kg^−1^), and ATMi + PARPi (5 mg kg^−1^ KU‐55933 + 25 mg kg^−1^ Olaparib). Scale bars: 100 µm. E) Violin plot of STAG2 mRNA expression level (log2(fpkm‐uq)) of normal and tumor tissues in the TCGA colorectal adenocarcinoma (COADREAD) cohort. Error bars indicate a standard error and *p*‐value was calculated using Student's *t*‐test. F) Kaplan–Meier curves depicting overall survival (OS) of patients with high and low STAG2 mRNA expression levels in the TCGA colorectal adenocarcinoma (COADREAD) cohort. Statistical analysis was performed using log‐rank test. G) Representative immunohistochemical images of low and high expression of STAG2 in the SYSUCC colorectal adenocarcinoma (COADREAD) cohort. Scale bars: 200 µm. H) Kaplan–Meier curves depicting overall survival (OS) of patients with high and low STAG2 protein expression levels in the SYSUCC colorectal adenocarcinoma (COADREAD) cohort. Statistical analysis was performed using log‐rank test.

Based on our findings that STAG2 regulates H4K20 methylation status via KMT5A to promote HR repair, we investigated the expression levels of H4K20me0, H4K20me1, and KMT5A in STAG2^WT^ and STAG2^KO^ tumors. Consistent with the results in vitro, STAG2^KO^ tumor lysates exhibited increased expression of KMT5A and H4K20me1 protein, but a marked reduction of H4K20me0 expression (**Figure** **6**A,B; Figure S6A, Supporting Information). Additionally, the DNA damage biomarker *γ*‐H2AX as well as the apoptosis marker cleaved Caspase 3 were highly expressed in STAG2^KO^ tumors after treatment with the combination of ATMi and PARPi (Figure 6C,D; Figure S6B, Supporting Information).

We next analyzed the data of various tumor types from the Cancer Genome Atlas (TCGA). Among the tumor types with a high frequency of STAG2 mutation, STAG2‐mutant uterine corpus endometrial carcinomas (UCEC) showed a significant increase in KMT5A expression compared to STAG2‐proficient UCECs (Figure S6C, Supporting Information). In stomach adenocarcinoma (STAD), lung adenocarcinoma (LUAD), and colon adenocarcinoma (COAD), STAG2‐mutant tumors exhibited a trend of high KMT5A expression compared with STAG2^WT^ tumors. (Figure S6D–F, Supporting Information). These data suggest that STAG2 deficiency upregulates KMT5A expression in human tumors and results in the accumulation of DNA damage and apoptosis when treated with the combination of ATMi and PARPi.

In the TCGA database, we found that the expression of STAG2 mRNA in tumor tissues was higher than that of normal colon tissue (Figure 6E). Importantly, colorectal adenocarcinoma (COADREAD) patients with high STAG2 expression had significantly worse overall survival than those with low STAG2 expression (Figure 6F). To confirm this finding, we collected a colorectal adenocarcinoma patient cohort from our institute and examined the expression of STAG2 in tumor tissues. Consistent with the data from TCGA, we observed that high expression of STAG2 relates to the adverse outcome of patients with colorectal cancer (Figure 6G,H). Aligned with our prior assertion, STAG2 emerges as a recurrently mutated gene across diverse cancer types, often accompanied by concurrent loss of expression. In view of this, we embarked upon a rigorous exploration to ascertain whether the prognostic significance associated with STAG2 mutations mirrored analogous trends. To this end, we meticulously conducted an analysis of STAG2 mutation data within comprehensive pan‐cancer datasets procured from TCGA. The data categorization involved a meticulous tripartite stratification, with the sample groups classified on the basis of STAG2 mutation status as wild‐type, amplification, or mutation (excluding amplification). Notably, the outcome of this meticulous inquiry unveils a consistent and robust pattern: diminished STAG2 expression, whether absent or attenuated, correlates unmistakably with improved patient outcomes (Figure S6G,H, Supporting Information). This compelling evidence proffers a dual dimension to STAG2's prognostic potential, wherein both STAG2 expression and mutation patterns collectively serve as potent biomarkers for prognostic evaluation.

## 3 Discussion

The genetic concept of synthetic lethality (SL) in tumor‐targeted therapeutics holds great promise but as yet remains to be elucidated. Human tumors with one dysfunctional DNA repair pathway may become hyper‐dependent on an alternative pathway for survival. PARP1 inhibitors have proven clinical efficacy for BRCA1/2‐mutated ovarian and breast cancers with deficient HR repair pathways.^[^ ^35^, ^36^, ^37^ ^]^ In the present study, we demonstrated that depletion of STAG2 results in hypersensitivity to ATM inhibitors in cellular and animal models. STAG2 regulates methyltransferase KMT5A expression to alter H4K20 methylation, which is required for the BRCA1‐BARD1 recruitment in HR repair. Loss of STAG2 leads to restoration of KMT5A expression and decrease in unmethylated H4K20, which causes the reduction of BRCA1 recruitments, DNA break repair, and eventually chromosomal abnormalities.

Mutations in cohesin and cohesin‐associated components are frequent characteristics of human tumors. More specifically, STAG2 mutation has been identified in a significant number of solid tumor types, including bladder, endometrial, stomach, and colorectal cancers.^[^ ^9^ ^]^ In accordance with genomic datasets of tumor patients (cBioPortal), ≈41% of STAG2 mutants are truncating mutations that are recognized as putative pathogenic mutations.^[^ ^41^ ^]^ SL provides a conceptual framework for developing targets that are traditionally undruggable in malignancies. The technological advances in high‐throughput screening have led to a rapid increase in the understanding of genetic interaction profiles and the ability to identify novel SL drug targets.^[^ ^42^ ^]^ Given our previous data showing sgRNAs directed against STAG2 conferred ATM inhibitor sensitivity,^[^ ^4^ ^]^ we further determined whether tumor cells with STAG2 depletion were susceptible to ATM inhibition. Here, we demonstrated that tumor cell lines with STAG2 defect are hypersensitive to ATM inhibitors as well as other DNA damage response‐related stimuli (e.g., irradiation, camptothecin, and cisplatin). The increased sensitivity in STAG2‐knockout tumors was characterized by increased DNA damage and chromosomal aberrations. Consistent with these findings, the loss function of STAG2 results in a stalled replication fork and sensitizes to ATR and PARP1 inhibitors due to an impaired DSB repair.^[^ ^22^, ^43^ ^]^ Taken together, these results highlight the importance of STAG2 signaling for efficient DNA repair following DNA damage.

STAG2 plays multiple cellular roles, many of which can impact DNA repair and genome stability.^[^ ^10^ ^]^ STAG2 is a critical mediator at the site of DNA replication that governs the interaction between the cohesin ring and replication‐related proteins.^[^ ^22^ ^]^ In addition to DNA replication, STAG2 also plays an important role in DNA DSB repair. STAG2‐mutant tumor cells result in a switch from STAG2 to STAG1‐cohesin complexes, differential cohesin dependence on DNA damage repair, and an increased sensitivity to PARPi.^[^ ^44^ ^]^ Moreover, STAG2‐mutated tumor cells are defective in HR repair upon depletion of STAG1, as evidenced by the HR‐defective score.^[^ ^40^ ^]^ Exactly what function of STAG2 is important with respect to HR repair remains at this point poorly understood. Our data showed that knockout of STAG2 results in decreased RAD51 foci and HR activities and BRCA1‐BARD1 chromatin enrichment, thereby impairing DSB HR repair.

Recently, mechanisms have been detailed for the recruitment and retention of BRCA1‐BARD1 at DSB sites, including interactions between the BRCA1‐A complex and K63‐linked Ub chains,^[^ ^45^, ^46^ ^]^ and the co‐occurrence of H2A K15‐Ub and H4K20me0.^[^ ^16^, ^47^, ^48^ ^]^ The recognition of H4K20me0 by BRCA1‐BARD1 contributes to the HR process and the recognition of H4K20me1 by 53BP1 contributes to the NHEJ process, respectively.^[^ ^16^, ^17^ ^]^ Unexpectedly, our data revealed that STAG2 knockout decreases the H4K20me0 level, the interaction between BARD1 and histone, and the accumulation of BRCA1 at the DNA damage site. Taken together, these findings highlight the important role of STAG2 in H4K20‐dependent HR repair. KMT5A has been suggested as a writer of histone methylation for H4K20 transformation.^[^ ^34^ ^]^ Thus, we reasoned that STAG2 may facilitate HR activity through the KMT5A‐mediated H4K20 methylation switch. We showed that STAG2 can inhibit the expression of KMT5A, which sustains the unmethylation status of H4K20; furthermore, reducing KMT5A expression can rescue the defect of HR (i.e., BRCA1 accumulation, DR‐GFP efficiency, sensitivity to PARPi) induced by STAG2 knockout, emphasizing the critical function of STAG2 in transcriptional regression in DNA DSB repair. Notably, the impaired HR by STAG2 deficiency increases the sensitivity to PARPi, consistent with previous reports.^[^ ^35^, ^36^, ^37^, ^38^ ^]^ More importantly, our data identify that knockout of STAG2 improves the synergistic effect of ATMi and PARPi, highlighting that loss of STAG2 is another condition that can further sensitize cells to the combination of PARP and ATM inhibitors.

STAG2 emerges as a gene subject to frequent mutations in the context of human cancers, exhibiting a noteworthy prevalence of mutational events alongside concurrent loss of expression. This functional aberration of STAG2 aligns with a prognosis that bears favorable implications, a conclusion substantiated by previous investigations.^[^ ^49^ ^]^ This intricate interplay assumes a pivotal role in underpinning the characteristic framework encompassing decreased recurrence and ameliorated clinical outcomes, evident within tumor settings characterized by STAG2 deficiency. Within the confines of our study, a rigorous examination of STAG2 expression patterns among patients grappling with colorectal cancer yielded an observation of paramount importance: heightened STAG2 expression markedly correlates with diminished patient outcomes. We posit the conjecture that elevated STAG2 expression fosters catalysis of homologous recombination, thereby conferring a consequential influence on the dynamics of tumor cell growth. This orchestrated modulation permeates through meticulous adjustments affecting genomic stability, transcriptional regulation, and the orchestrated response to DNA damage. The convergence of these findings fuels the compelling proposition that STAG2's functional role encompasses potential oncogenic attributes or the capacity to modulate drug resistance mechanisms. In light of these intriguing prospects, a logical extension of investigating STAG2's potential as a biomarker lies in the exploration of targeted inhibitors or therapeutic interventions specific to STAG2. This untapped avenue stands poised to offer innovative strategies, notably for patients characterized by heightened STAG2 expression levels.

Altogether, our results demonstrate that defects in STAG2 cause DNA damage and reduce HR by restoring the expression of KMT5A and the switch of H4K20me to H4K20me1, which is required for the recruitment of BRCA1‐BARD1 to chromatin. More importantly, loss of STAG2 causes hypersensitivity to ATMi, PARPi, and the combination of the inhibitors. Since STAG2 mutations occur in a variety of tumors, the combination strategy may have significant implications for developing treatment.

## 4 Experimental Section

### Cell Culture and Transfections

DLD1, HCT116, UMUC3, SNU719, AGS, H460, and T24 were cultured in Roswell Park Memorial Institute (RPMI) 1640 medium with 10% fetal bovine serum (FBS) (Gibco, NY, USA). HEC‐1A, ISHIKAWA, A549, U2OS, HeLa, and HEK293T cells were cultured in Dulbecco's modified Eagle's medium (DMEM) with 10% FBS (Gibco, NY, USA) and penicillin‐streptomycin (1%). All cell lines were purchased from the American Type Culture Collection (ATCC) in 2017. All cultures were maintained in an incubator at 37 °C (Thermo Fisher Scientific, Waltham, MA, USA) with 5% CO_2_ in culture dishes (JET BIOFIL, Guangzhou, China), and tested negative for mycoplasma contamination. All transfections were performed using Lipofectamine 2000 (Invitrogen, Carlsbad, CA, USA) according to the manufacturer's instructions.

### Generation of Knockout Cell Lines with Crispr‐Cas9

To generate T24, A549, NCI‐H460, HeLa, and U2OS cells expressing the indicated sgRNAs, the oligonucleotides described in Table S1 (Supporting Information) were cloned into the pLenti‐CRISPR V2 viral vector. Cells were transfected with Cas9‐gRNA plasmids and selected using puromycin 48 h later, then cultured for approximately three weeks for colonies. The clones were tested by western blot for the efficiency of knockouts.

### siRNA Transfection

For siRNA transfection, cells were seeded at ≈30% confluence into 6‐well plates and transfected with a specific siRNA duplex using Lipofectamine 2000 (Invitrogen, Carlsbad, CA, USA) for 48 h following the manufacturer's instructions. The sequences of the siRNAs used in this study are shown in Table S1 (Supporting Information).

### Colony Formation Assay

Cells were seeded at a concentration of 500–1000 cells per well into six‐well plates or 100–300 cells per well into 24‐well plates. After 24 h, 12 different tumor cell lines were treated with indicated compounds continuously ranging from 10 to 14 days, depending on their distinguished growth characteristics. Colonies were fixed with fixation solution (methanol:acetic acid = 5:1 v/v) at room temperature for 20 min and then stained with a solution of 0.5% crystal violet in methanol for two hours.

### Analysis of HR and NHEJ Activities

The efficiency of HR and NHEJ were measured using the U2OS DR‐GFP and EJ5‐GFP reporter cell lines, as previously described.^[^ ^50^ ^]^ Briefly, 24 h before infection with I‐Sce‐I adenovirus, U2OS cells with either GFP expression cassette were transfected with the indicated siRNA. The activity of HR and NHEJ was measured by flow cytometric quantification of viable GFP^+^ cells 72 h after I‐Sce‐I adenovirus infection.

### Antibodies and Chemicals

The antibodies used in this study were as follows: STAG2 (Cell Signaling Technology (CST) 5882S, IF, 1:200; IB, 1:1000; IHC, 1:100), BRCA1 (CST 9010S, IB, 1:1000; Santa Cruz 2996T, IF, 1:50), BARD1 (Bethyl Laboratories A300‐263A‐T, IB, 1:1000), *γ*‐H2AX (CST 9718S, IB, 1:1000; IF, 1:200; IHC, 1:100), RAD51 (Abcam ab133534, IF, 1:200; IB, 1:10 000), 53BP1 (CST 4937S, IF, 1:200; IB, 1:1000), RPA32 (CST 2208S, IF, 1:200; IB, 1:1000), RAD21 (CST 4321S, IB,1:1000), H4 (Abcam ab177188, IB, 1:1000), H4K20me0 (Abcam ab227804, IB, 1:1000; IHC, 1:100), H4K20me1 (Abcam ab177188, IB, 1:1000; IHC, 1:100), KMT5A (CST 2996T, IF, 1:100; IB, 1:1000), H3 (Abcam Ab1791, IB, 1:1000), Lamin B1 (Abcam Ab16048, IB, 1:1000), FLAG (CST 14793S, IB, 1:1000; IP, 1:50; CUT&Tag, 1:50), cleaved‐Caspase‐3 (CST 9661T, IB, 1:1000; IHC, 1:200), *α*‐tubulin (CST 2144S, IB, 1:1000), GAPDH (Proteintech 60004‐1‐Ig, IB, 1:1000).

The chemicals used in this study were as follows: KU‐60019, KU‐55933, and AZD2281 (Olaparib) were purchased from Selleck Chemistry. Colchicine (ST1173), Crystal violet (C0121), and Giemsa (C0133) were purchased from Beyotime.

### Western Blotting

Cell lysis was performed using a lysis buffer (300 mm NaCl, 50 mm Tris‐Cl, 1 mm EDTA, 0.5% NP‐40), and the lysates were separated on denaturing Nu‐page (Invitrogen) polyacrylamide gels before being transferred onto nitrocellulose membranes. After blocking with 5% milk in TBST, the membranes were probed with primary and secondary antibodies and detected with chemiluminescence (Tanon, 180–5001).

### Co‐Immunoprecipitation Assay (Co‐IP)

Co‐IPs were performed with the Dynabeads Protein G (Invitrogen, 10004D) according to the manufacturer's instructions. In brief, Dynabeads (1.5 mg) was conjugated with antibodies overnight at 4 °C. Antibodies were used as indicator on the suppliers’ datasheets for each antibody. Next day, the total cell lysates and the antibody‐conjugated Dynabeads were incubated overnight at 4 °C with shaking. After three times washing with PBS containing 0.1% Tween, the beads were boiled at 95 °C for 5 min in the 6×protein loading buffer (Beyotime, P0015) and the supernatant was collected for future Western blotting analysis.

### Xenograft Models

The study is compliant with all relevant ethical regulations regarding animal research and was approved by the Sun Yat‐Sen University Animal Care and Use Committee. Athymic nude mice (BALB/c nu/nu, 5 weeks old) were purchased from Vital River Laboratories (Beijing, China), and housed under standard conditions in the animal care facility at the Center of Experimental Animals of Sun Yat‐sen University. For the subcutaneous xenograft model, control and experimental DLD1 (2 × 10^6^) were suspended in 100 µL PBS and then injected subcutaneously into the flanks of the nude mice (n = 6). When the tumor sizes reached 3–4 mm, mice were intraperitoneally injected with the indicated drugs once every three days. The dosages of drugs were as follows: ATMi, 10 mg kg^−1^ KU‐55933; PARPi, 50 mg kg^−1^ AZD2281; combination experiment: single 5 mg kg^−1^ KU‐55933, single 25 mg kg^−1^ AZD2281, or combined 5 mg kg^−1^ KU‐55933 + 25 mg kg^−1^ AZD2281. The dynamic assessment of tumor growth was meticulously conducted at three‐day intervals over a span of 2 weeks. Subsequently, the quantification of tumor volumes was executed utilizing the equation (length x width^2^)/2. After 2 weeks, the mice were euthanized and the tumors were harvested, fixed, and paraffin‐embedded for further analysis.

### Calculation of Coefficient of Drug Interaction

To determine drug interactions between two drugs (i.e., additive, synergistic, or antagonistic), we calculated the coefficient of drug interaction (CDI) by the formula CDI = AB/(A × B), where AB is the ratio of the combination to the control, and A or B is the ratio of the single drug contrasted to the control. Thus, a CDI = 1.0 indicates an additive interaction, a CDI <1.0 indicates a synergistic interaction and a CDI >1.0 indicates an antagonistic interaction. Further, CDI <0.7 indicates a good synergistic interaction.

### Immunohistochemistry (IHC) and Histological Score (H‐score)

The IHC staining process involved a methodical sequence of steps. Initially, the CRC specimens were subjected to deparaffinization and hydration, followed by an incubation period with 3% H_2_O_2_ for 10 min to effectively neutralize endogenous peroxidase activity. Subsequently, antigen retrieval was accomplished through a 90‐s steaming process using citrate buffer (pH 6.0, P0081, Beyotime, China). Following this, the specimens were subjected to a 30‐min blockade using 5% bovine serum albumin, subsequent to which, they were incubated overnight at a temperature of 4 °C with a primary rabbit anti‐human antibody (anti‐STAG2 antibody, 5882S, CST). A secondary phase ensued, involving the incubation of the specimens with a goat anti‐rabbit horseradish peroxidase‐conjugated secondary antibody, with this step taking place for a duration of 30 min at 37 °C. Post three thorough washes, the specimens were subjected to 3,3′‐diaminobenzidine incubation, followed by counterstaining with hematoxylin. The evaluation of histological scores (H‐scores) was conducted utilizing a calculated framework based on distinct staining intensities. The H‐score for each sample was determined using the formula: 1 × (% weak staining) + 2 × (% moderate staining) + 3 × (% strong staining). Consequently, the resultant H‐score values spanned a range of 0–300. The evaluation of the slides was undertaken in an impartial manner by two seasoned pathologists, whose evaluation was conducted in isolation from the clinical parameters.

### Neutral Comet Assay

Neutral comet assay can mainly detect double‐strand breaks (DSBs) at the individual cell level.^[^ ^51^ ^]^ In brief, cells were trypsinized and suspended in cold 1×PBS (Ca^2+^ and Mg^2+^ free), mixed with low melting agarose (Trevigen) at a ratio of 1:10 (v/v), and immediately plated onto Cometslide (Trevigen). Neutral electrophoresis was run at 25 V for 30 min in the electrophoresis system. Cell comets were imaged using a fluorescence microscope.

### Chromosomal Abnormality Analysis

U2OS cells transfected with the indicated sgRNA were incubated with or without 5 µM KU‐60019 for 24 h. Cells were treated with 100 ng mL^−1^ of colchicine for 2 h, followed by a hypotonic solution (0.075 m KCl) for 20 min and fixed in 3:1 methanol/acetic acid. After being stained with Giemsa stain, at least 30 metaphase spreads were counted for aberrations. The relative number of chromosomal aberrations was calculated normalized to empty vector control or wild‐type.

### Immunofluorescence Assays

Cells were plated on coverslips, washed once with PBS, and fixed with 4% paraformaldehyde for 10 min on ice. Then, fixed cells were incubated with 0.5% Triton X‐100 for 30 min, followed by blocking with 3% BSA for at least 30 min and incubation with primary antibody (diluted in 3% BSA) overnight at 4 or 37 °C for 2 h. Following incubation, coverslips were washed three times with PBST, and incubated with a secondary antibody (Alexa Fluor, Life Technologies) for 1 h at room temperature. Coverslips were then mounted with DAPI medium and visualized under the fluorescence microscope. At least 100 cells were counted for each sample.

### Subcellular Fractionation

Following the manufacturer's instructions, a subcellular fractionation kit (CST, 78840) was used to isolate subcellular fractions. The protein lysates of each fraction were analyzed by western blotting using indicated antibodies.

### CUT&Tag

The cleavage under targets and tagmentation (CUT&Tag) experiments were performed using the vector or 3×FLAG‐STAG2 restoring STAG2 knockout U2OS cells by the Frasergen Company (Wuhan, China).^[^ ^52^ ^]^ Concanavalin A‐attached magnetic beads, which were used to bind the cells, then the cell membrane was permeabilized with digitonin. The primary antibody of FLAG (CST, 14793S) and the corresponding secondary antibody were incubated on a rotating platform overnight at 4 °C. Then, the transposon fused with protein A/G accurately targeted and cleaved the DNA sequence near the STAG2 protein. Next, adaptor sequences were added to both ends of the cleaved fragments during transposon cleavage. To PCR amplify libraries, cleaved DNA was mixed with a universal i5 and a uniquely barcoded i7 primer, using a different barcode for each sample. Following PCR amplification, the sequence can be directly used for high‐throughput sequencing.

### TCGA Data Acquisition and Analysis

The Cancer Genome Altas (TCGA) mutation and mRNA expression data were obtained from cBioportal (https://www.cbioportal.org/). The samples building GSEA analysis were confined to these primary diseases: colon adenocarcinoma, bladder urothelial carcinoma, lung squamous cell carcinoma, lung adenocarcinoma, stomach adenocarcinoma, rectum adenocarcinoma, uterine corpus endometrioid carcinoma. Patients were grouped into high‐ and low‐STAG2 expressions according to the optimal cut‐point of continuous variables determined using the surcutpoint function by the survminer package. The Kaplan–Meier curves were used to plot the survival, and the log‐rank test to estimate the difference in the survival curves.

### Patients and Tissue Specimens

Paraffin samples were collected from October 2012 to December 2014 by the Department of Pathology, Sun Yat‐sen University Cancer Center. The inclusion criteria for selecting colorectal adenocarcinoma (COADREAD) cases included: precise imaging and pathological diagnosis, and access to complete follow‐up data. Cases were excluded if patients had previously received anti‐tumor treatment. All samples were collected with the informed consent of patients under institutional review board‐approved protocols and stored at −80 °C in the SYSUCC Bio‐bank until use. All samples used in this study were approved by the Committees for Ethical Review of Research Involving Human Subjects at the Sun Yat‐Sen University Cancer Center, and this study was performed in accordance with the Declaration of Helsinki. Written informed consent was obtained from the patients before the study began.

### Statistical Analysis

Statistical analysis was performed using the Prism 8 GraphPad software, SPSS 20.0 software (SPSS, Chicago, IL, USA), and R software (version 4.1.0). The statistical significance between the two groups was compared using the two‐tailed Student's *t*‐test. ANOVA was used for multiple comparisons of three or more independent groups. Data were presented as mean ± SEM, and a *p*‐value <0.05 was considered statistically significant.

## Conflict of Interest

The authors declare no conflict of interest.

## Author Contributions

J.Z., R.C.N., Z.P.H., and X.X.C. contributed equally to this work. J.Z. and R.C.N. conceived the study, performed experiments, analyzed data, and wrote the manuscript. Z.P.H. and X.X.C. performed the animal experiments and analyzed the data. J.W.C., W.P.L., Y.X.Y., and Z.C.X. performed the IHC analysis and analyzed the data. T.C.Z., J.J.X., and Y.C.Z. helped in performing the cell and molecular biology experiment. X.W. and P.L. helped analyze the clinical data. D.X. provided critical advice on the study and contributed to writing the manuscript. D.X., A.D.D., and M.Y.C. conceived the study and wrote the manuscript.

## Supporting information

Supporting Information

## Data Availability

The data that support the findings of this study are available on request from the corresponding author. The data are not publicly available due to privacy or ethical restrictions.
