# Supplementary material for: STAG2 Regulates Homologous Recombination Repair and Sensitivity to ATM Inhibition
Source: Adv Sci (Weinh). 2023 Nov 20;10(36):2302494. doi: 10.1002/advs.202302494 (PMC10754142; doi:10.1002/advs.202302494)
Supplement: Supplementary file 1 — Supporting Information [file ADVS-10-2302494-s001.pdf]

ADVANCED SCIENCE
Open Access

Supporting Information

for *Adv. Sci.*, DOI 10.1002/advs.202302494

STAG2 Regulates Homologous Recombination Repair and Sensitivity to ATM Inhibition

Jie Zhou, Run-Cong Nie, Zhang-Ping He, Xiao-Xia Cai, Jie-Wei Chen, Wen-ping Lin, Yi-Xin Yin, Zhi-Cheng Xiang, Tian-Chen Zhu, Juan-Juan Xie, You-Cheng Zhang, Xin Wang, Peng Lin, Dan Xie, Alan D D'Andrea* and Mu-Yan Cai**

Supplemental information

Supplementary Figures

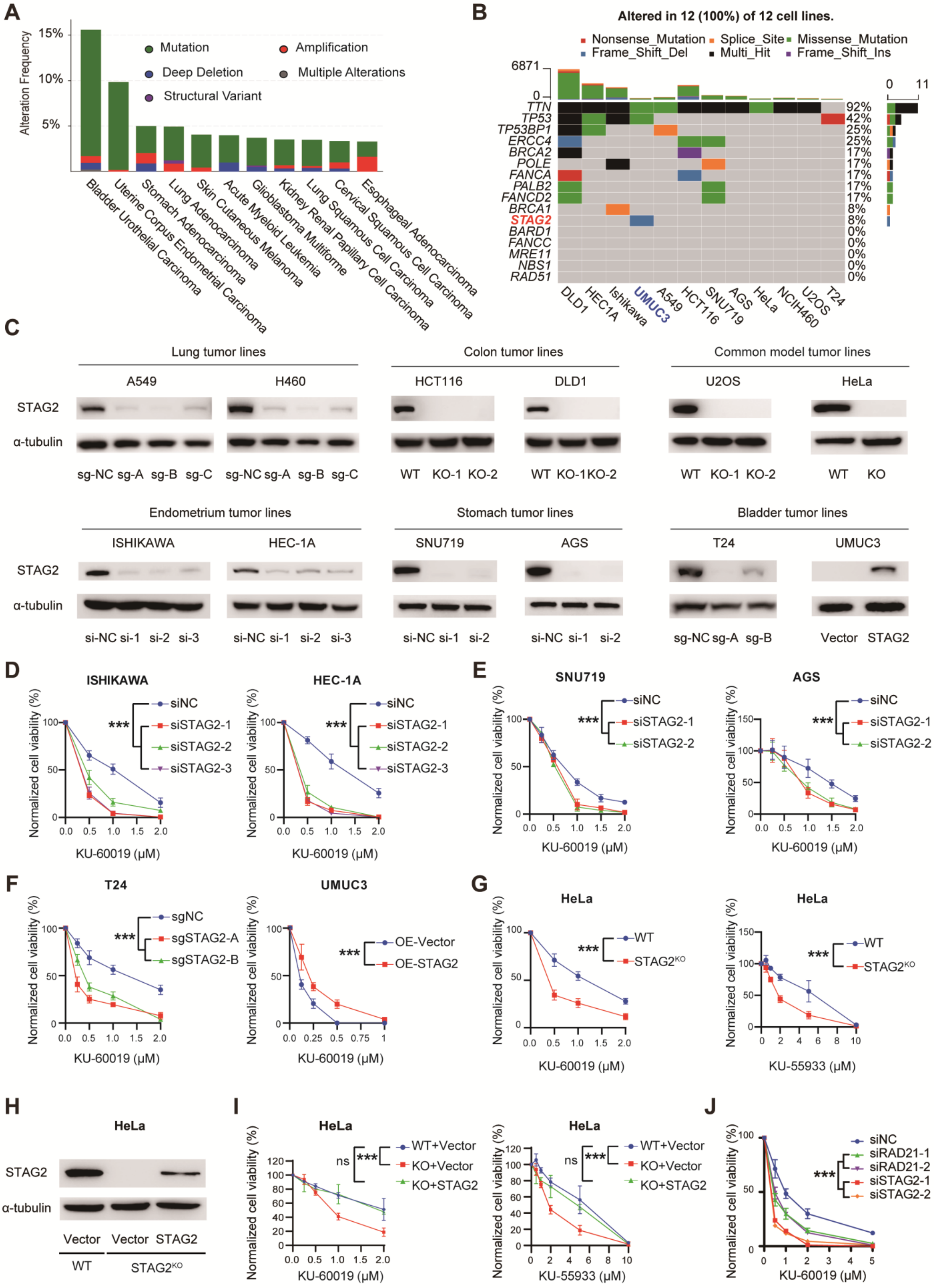

Figure S1. STAG2 is synthetically lethal with ATM inhibitor in multiple cancers, related to Figure 1.
(A) Bar chart showing the summary of STAG2 genomic alterations across various cancer types in TCGA.
(B) Mutation information of 12 tumor cell lines used in this study.
(C) Western blot showing the efficiency of inference, and knockout by siRNA and sgRNA via CRIPSR-Cas9 in 12 cell lines, overexpression by the lentiviral overexpression plasmid.
(D-E) Clonogenic survival assays of si-Control and si-STAG2 endometrial cancer cells (D) and gastric carcinoma cells (E) after treatment with ATM inhibitor (KU-60019) . -1,-2,-3 indicate three independent siRNAs.
(F) Left: Clonogenic survival assay of control and KO bladder cancer cells after treatment with ATM inhibitor (KU-60019) following transduction with STAG2-targeting sgRNAs. -A, -B indicate two independent sgRNAs. Right: Clonogenic survival assay of STAG2-mutated bladder cancer cells treated with ATM inhibitor (KU-60019) with or without STAG2 overexpression.
(G) Clonogenic survival assays of HeLa cells after treatment with the two ATM inhibitor (left panel: KU-60019, right panel: KU-55933) in stable clones of STAG2 knockout via CRIPSR-Cas9.
(H-I) Western blot (G) and Clonogenic survival assays (H) detecting the efficiency of STAG2 rescue and its impact after treatment with two ATMi (left: KU-60019, right: KU-55933) in HeLa cells.
(J) Clonogenic survival assays of U2OS cells with STAG2 or RAD21 knockdown and treated with ATMi KU-60019.
Data are shown as mean ± SEM. Statistical analysis was performed using one-way (J) and two-way ANOVA (D, E, F, G and I). ns, not significant, *$p<0.05$, **$p<0.01$, ***$p<0.001$.

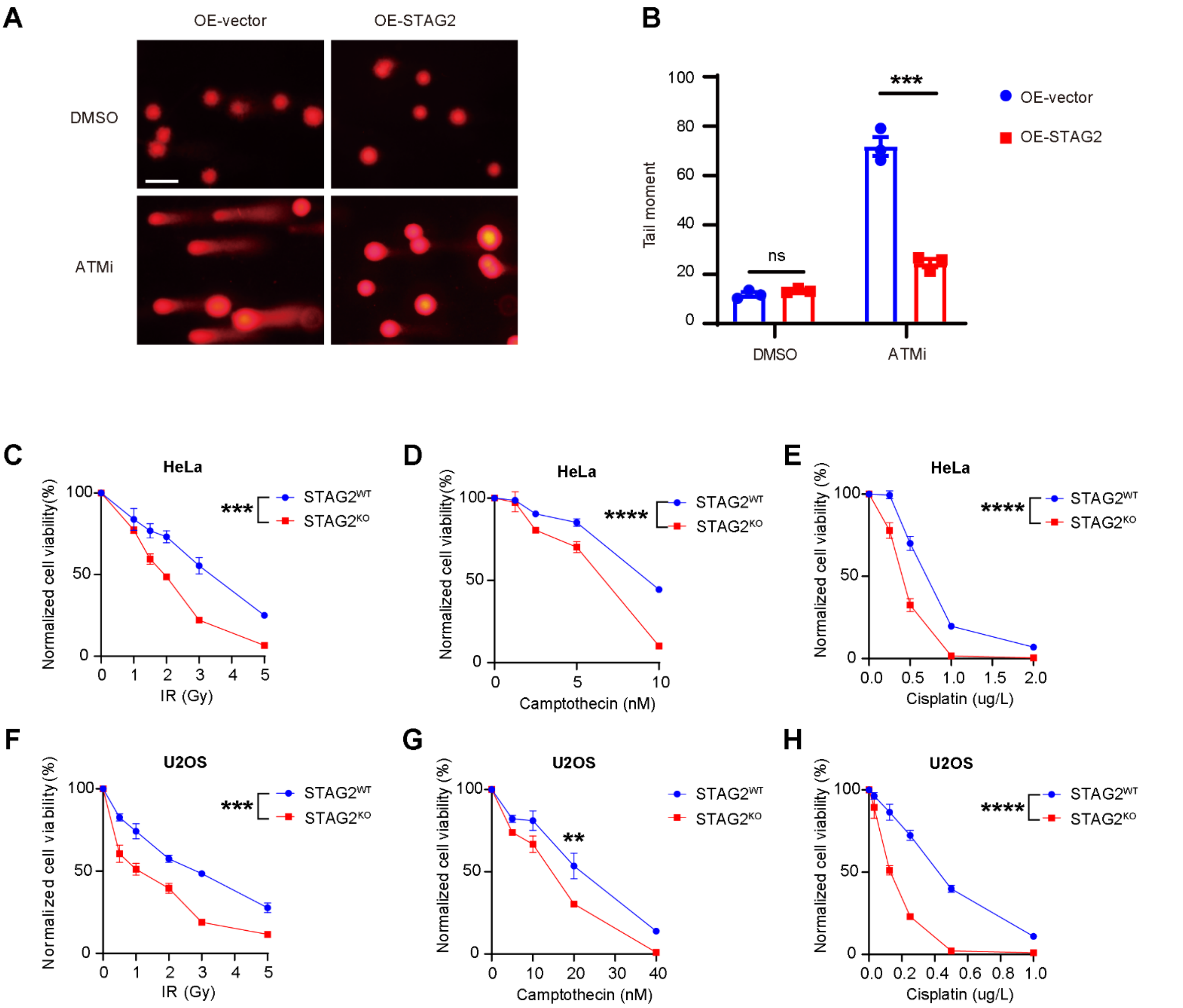

Figure S2. STAG2 deficiency increases the sensitivity of multiple DDR related stimulus.

(A) Representative images of neutral comets in with or without STAG2 overexpression UMUC3 cells after treatment with DMSO or KU-60019 (5 μM) for 24 hours. Scale bar: 100 μm.

(B) Percentage of the comet tail moment. At least 50 cells were counted in each condition. Data are shown as mean ± SEM, and were analyzed by Student's t test. ns: not significance, ns: not significance,***p<0.001.

(C-E) Clonogenic survival assays of wildtype (Ctrl) and STAG2^{KO} HeLa cells after treatment with three DDR induced stimulus: irradiation (C), camptothecin (D), and cisplatin (E).

(F-H) Clonogenic survival assays of wildtype (Ctrl) and STAG2^{KO} U2OS cells after treatment with three DDR induced stimulus: irradiation (F), camptothecin (G) and cisplatin (H). Data are shown as mean ± SEM, were analyzed two-way ANOVA test. **p<0.01, ***p<0.001, ****p<0.0001.

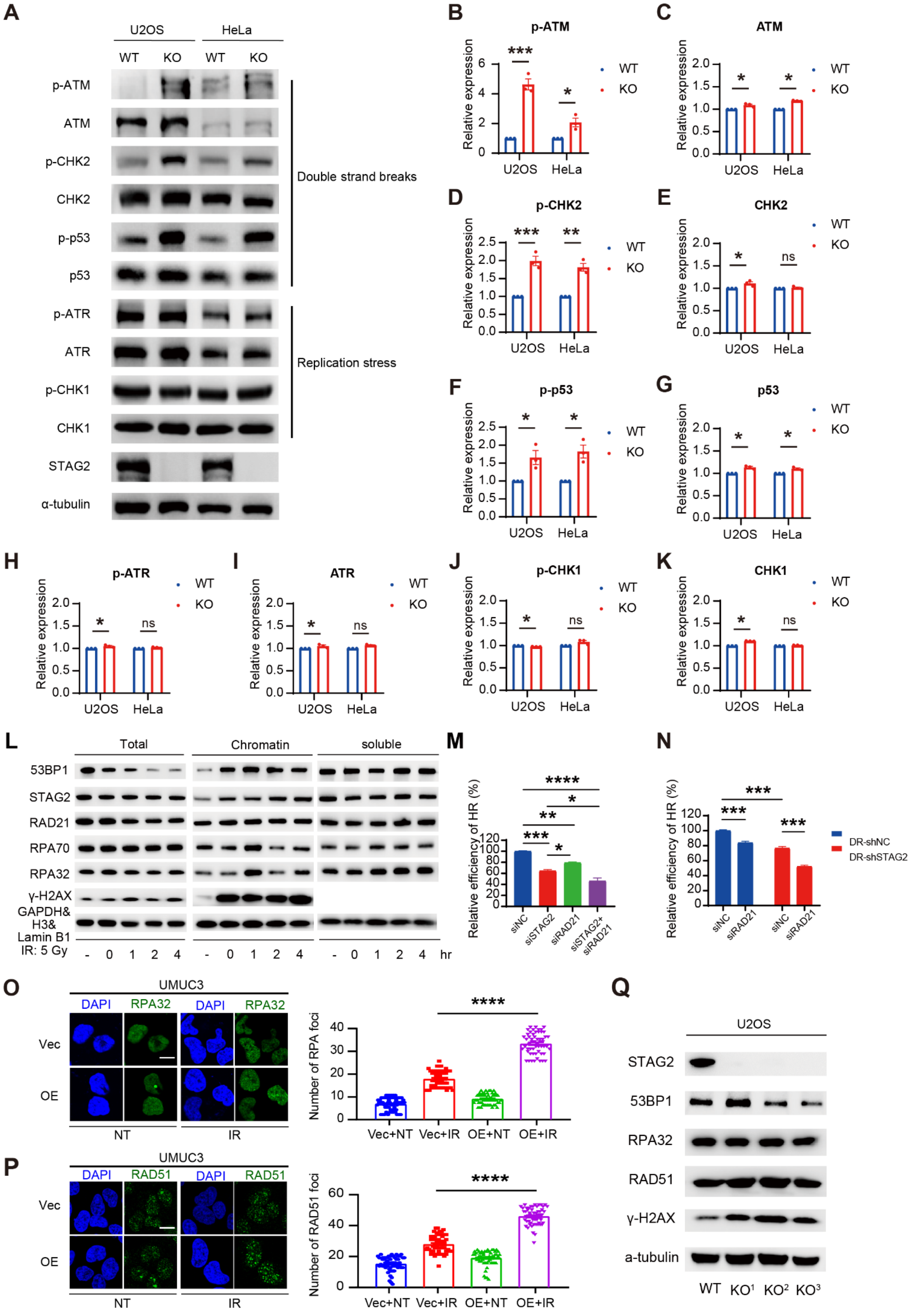

Figure S3. Knock out of STAG2 shows a minor impact in replication fork progression, and influences the DSB without influencing the expression of DSB proteins, related to Figure 3.

(A) Western blot of indicated protein expression in wildtype (WT) and STAG2 knockout (KO) U2OS and HeLa cells. **(B-K)** Quantification of the gray values of indicated bands in panel A.
(L) Western blot of the indicated proteins in subcellular fractions 0, 1, 2, and 4 hours after treatment or untreated (-) with irradiation (5 Gy) in U2OS cells. GAPDH was used as the controls for the total cell lysates. Histone H3 was used as the controls for the nucleus chromatin proteins. Lamin B1 was used as the controls for the nucleus soluble proteins
(M) Quantification of HR efficiency in DR-GFP cells after transduction with si-NC, si-STAG2 and si-RAD21.
(N) Quantification of HR efficiency in sh-NC or sh-STAG2 DR-GFP cells after transduction with si-NC and si-RAD21.
(**O-P**) Immunofluorescence images and quantification of RPA32 (O), RAD51 (P) foci 4 hours after treatment untreated (NT) or treated with irradiation (IR, 5 Gy) in transducing with empty vector (Vec) or over-expressing STAG2 (OE) UMUC3 cells. At least 50 nuclei were counted in each condition.
(Q) Western blot of indicated protein expression in wildtype (WT) and STAG2 knockout (KO) U2OS cells.
Data are shown as mean ± SEM, and were analyzed by Student's t test (B-K) and one-way ANOVA (O). ns: not significance, $^{*}p<0.05$, $^{**}p<0.01$, $^{***}p<0.001$. $^{****}p < 0.0001$.

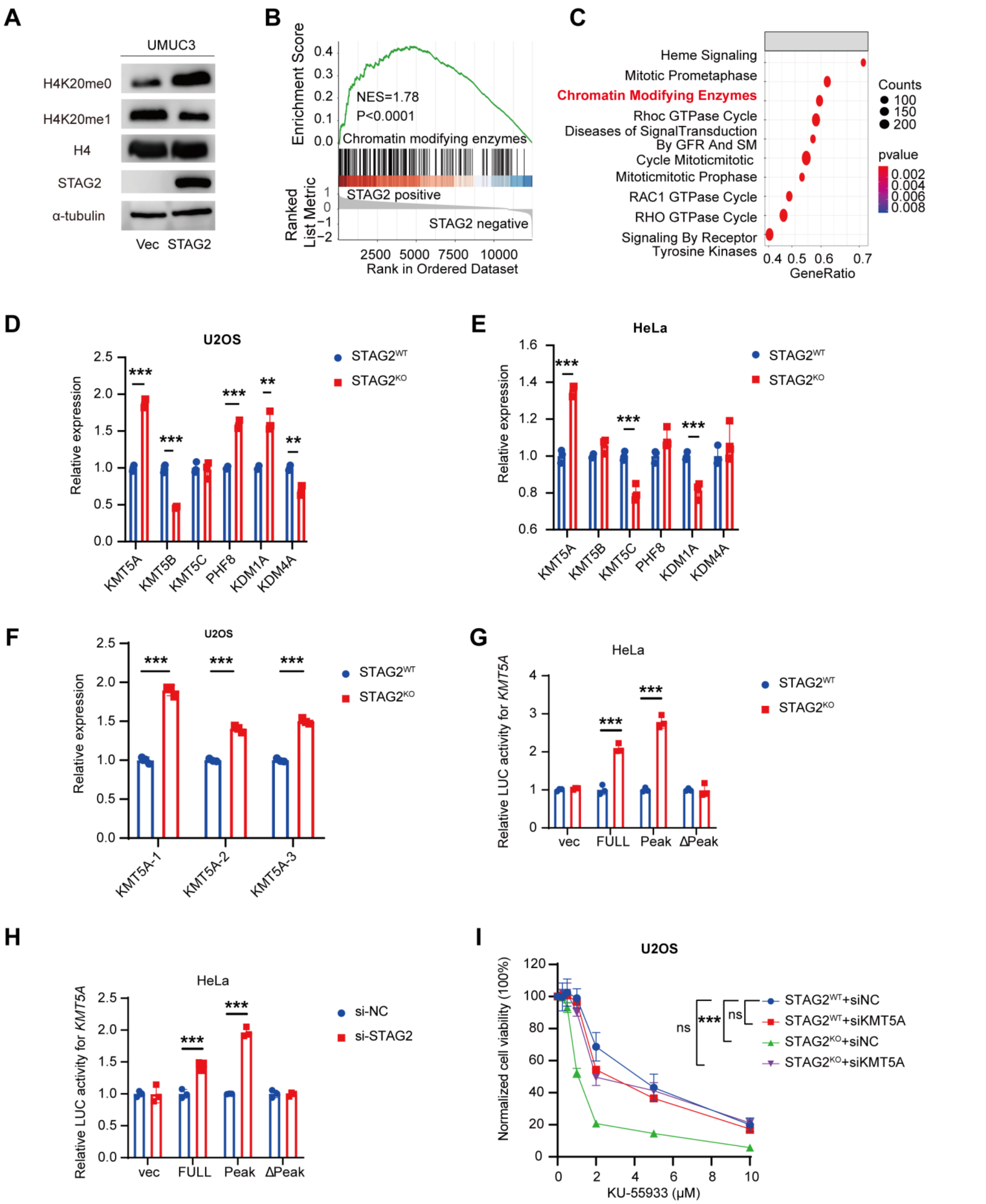

Figure S4. Knockout of STAG2 increases the expression of KMT5A, and the inference of KMT5A can restore the sensitivity of ATMi in STAG2^{KO} cell, related to Figure 4.

(A) Western blot of the indicated proteins from transducing with empty vector (Vec) or over-expressing STAG2 (OE-STAG2) plasmid UMUC3 cells.

(B) GSEA analysis of chromatin modifying enzymes pathway between samples with high and low STAG2 expression from TCGA.

(C) A Bubble graph showing the GO enrichment of differential expression analysis in high STAG2 expression from TCGA.   GFR: Growth Factor Receptors, SM: Second Messengers.
(D-E) Relative mRNA expression of indicated molecules in $STAG2^{WT}$ and $STAG2^{KO}$ U2OS (D) and HeLa (E) cells.
(F) Relative mRNA expression of KMT5A identified by three primers in $STAG2^{WT}$ and $STAG2^{KO}$ U2OS cells.
(G) Relative values of luciferase (LUC) activity measured by after transfection of different reporter plasmids in wild-type ($STAG2^{WT}$) or STAG2 knockout ($STAG2^{KO}$) HeLa cells (Relative to $STAG2^{WT}$ each group).
(H) Relative values of luciferase (LUC) activity measured by after transfection of different reporter plasmids in HeLa cells following by si-NC or si-STAG2 (Relative to si-NC each group).
(I) Clonogenic survival assays of $STAG2^{WT}$ and $STAG2^{KO}$ U2OS cells treated with siRNA negative control and si-KMT5A, and treated with DMSO or ATMi (KU-55933).
Data are shown as mean ± SEM, and were analyzed Student's t test (C-D), and two-way ANOVA test (F). ns, not significant, $^{**}p<0.01$, $^{***}p < 0.001$.

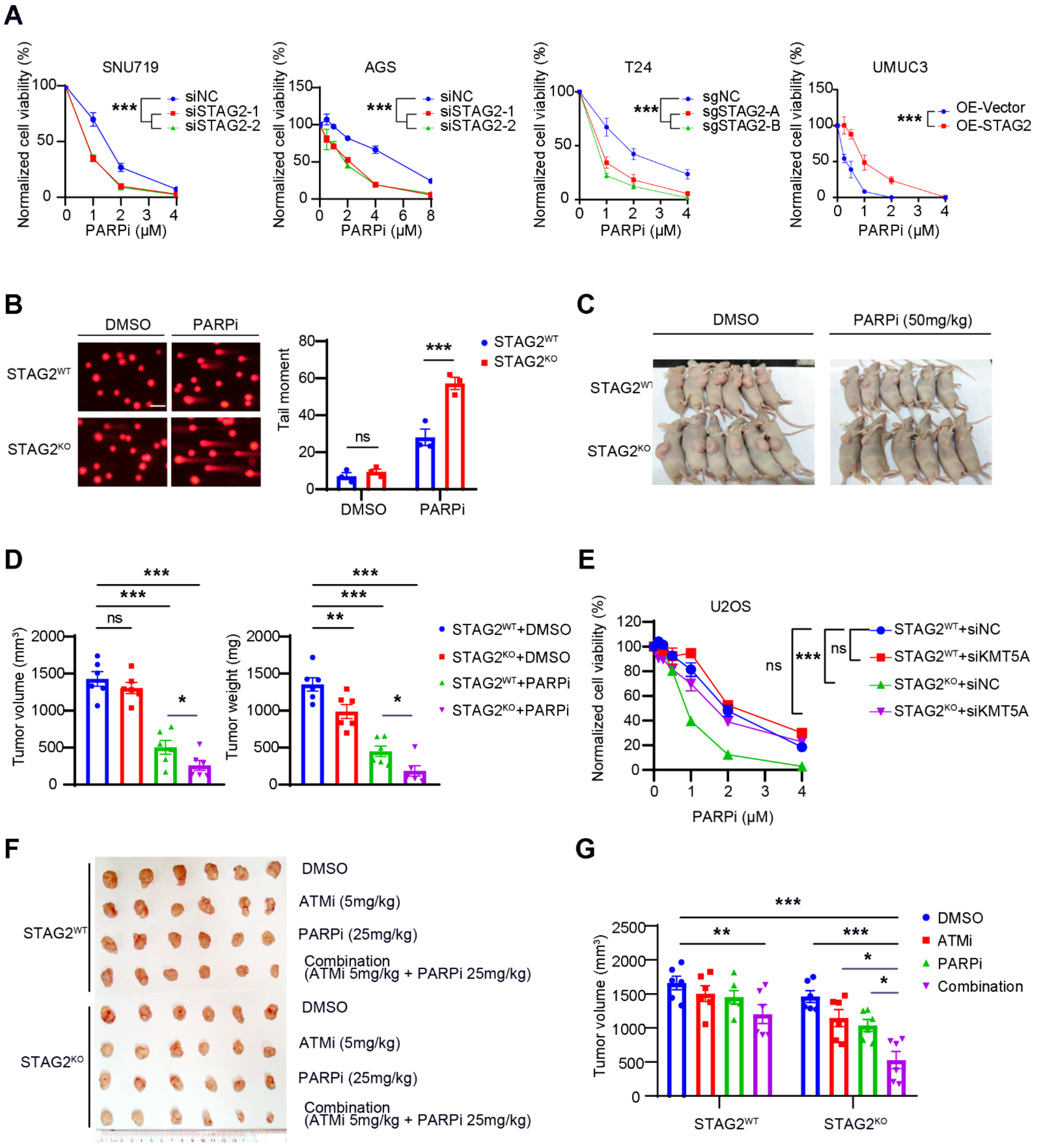

Figure S5. STAG2 deficiency increases the DSBs induced by PARP inhibitor, and increases the efficacy of ATMi and PARPi combination *in vivo*, related to Figure 5.

(A) Clonogenic survival assays of stomach (SNU719 and AGS) and bladder (T24 and UMUC3) cancer cells treatment with PARP inhibitor (PARPi, Olaparib) after si-STAG2, or sg-STAG2, or STAG2 overexpression.

(B) Left: Representative images of neutral comets in STAG2^{WT} and STAG2^{KO} U2OS cells after treatment with DMSO or PARP inhibitor (10 μM) for 24 hours. Scale bar: 100 μm. Right: Percentage of the comet tail moment. At least 50 cells were counted in each condition.
(C) Images of the mice sacrificed after the last volume measurement of tumors, related to **Figure 5B-C**.
(D) Tumor weights and volumes of xenograft tumors formed in nude mice (n = 6). Day 16 tumor weights (Left panel), and volumes (right panel) are shown, related to Figure **5B-C**.
(E) Clonogenic survival assays of STAG2^{WT} and STAG2^{KO} U2OS cells treated with si-NC and si-KMT5A, and treated with DMSO or PARPi (Olaparib), related to **Figure 5D**.
(F-G) Tumor images and weight of xenograft tumors formed in nude mice (n = 6 for each group). Day 16 tumor image (F) and tumor volumes (G) are shown, related to Figure **5G-H**.
Data are shown as mean ± SEM, and were analyzed by Student's t test (B), two-way ANOVA test (A and E), one-way ANOVA test (D and G). ns, not significant, *p<0.05, **p<0.01 ,***p<0.001.

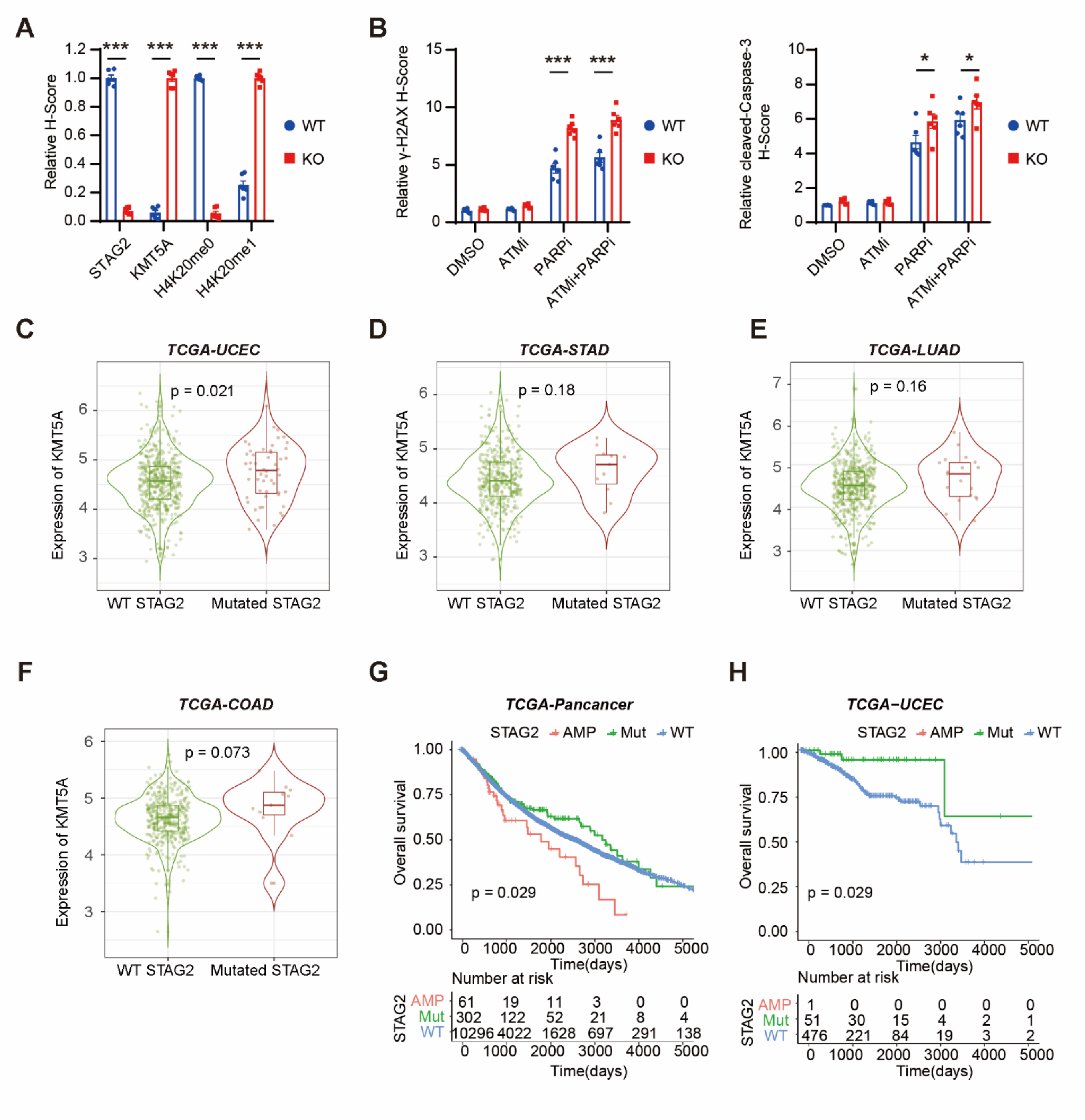

Figure S6. STAG2 mutation may correlate to high KMT5A expression in multiple cancers, and COAD of different MSS status with different STAG2 expression indicate distinct clinical outcome, related to Figure 6.

(A) Quantification of histologic score (H score) of indicated markers from six individual mice in wildtype (WT) and STAG2 knockout (KO) groups treatment with DMSO, related to **Figure 6A**.

(B) Quantification of histologic score (H score) of indicated markers from six individual mice in wildtype (WT) and STAG2 knockout (KO) groups with different treatment as indicated, related to **Figure 6B**.

(C-F) KMT5A (SETD8) mRNA expression of wildtype (WT) STAG2 and mutated STAG2 groups across multiple cancer types from TCGA datasets. Error bars indicate standard error and p-values were calculated using Mann Whitney Wilcoxon (MWW) test. UCEC: Uterine Corpus Endometrial Carcinoma; STAD: Stomach adenocarcinoma; LUAD: Lung adenocarcinoma; COAD: Colon adenocarcinoma.

Data are shown as mean ± SEM, and were analyzed by Student's t test. (A and B). *p<0.05, ***p<0.001.

(G-H) Kaplan-Meier curves depicting overall survival (OS) of patients with different mutation state of STAG2 in the TCGA. WT: wild-type, AMP: amplification, and Mut: mutation (excluding amplification). (G) Pancancer: pan-cancer dataset. (H) UCEC: Uterine Corpus Endometrial Carcinoma datasets. Statistical analysis was performed using log-rank test.

Supplementary Table

Table S1 The oligonucleotides used in this study

**si-RNA**		
STAG2-1	GCAGAAATGTTTAGACATA	
STAG2-2	GCAGTGTACTCACTATGTA	
STAG2-3	GCAAGAGAGTGCTCTGATT	
RAD21-1	GCCTGATAGTCCTGATTCA	
RAD21-2	AGAGGAAGCTAATTGTTGA	
BRCA1	CTACTCATGTTGTTATGAA	
53BP1	GGACAAGTCTCTCAGCTAT	
KMT5A	GGATGCAACTAGAGAGACA	
**sg-RNA**	Forward	Reverse
STAG2-A	TTGGAAAACGAGCCAATGAG	CTCATTGGCTCGTTTTCCAA
STAG2-B	ATACCTTGTGGATAGCATGT	ACATGCTATCCACAAGGTAT
STAG2-C	AGTCCCACATGCTATCCACA	TGTGGATAGCATGTGGGACT
**RT-PCR primer**	Forward	Reverse
KMT5A (-1)	GGAATGCGTTTCCCCCTTCA	TTTTTCAGGGCTTGCTTGGC
KMT5A-2	ACGTATTTACCGGGCAGTCA	TGGTTTCCCCTGGCATTTGA
KMT5A-3	ACGGCGTACCTCACCTCATCC	TGCGGTCCCCATAGTCATACAG G
KMT5B	TGAGAGGTGCTCATTCGCAG	TGGACATGCCTGTGTCACTC
KMT5C	CATGTACTCAACCCGCAAGC	GCCGTAGAAGCATGTCACCT
KDM4A	GACTGTTCGAGAGTTCCGCA	TCAGTGTGCCAAGCAAAGGA
PHF8	GGCTGCTGACATTGACCTCT	CATGCCCAACCCATCCTTCT
KDM1A	TTTGGAAGCCAGGGATCGTG	CTTTCTCTTTAGGAACCTTGACA GT
**PCR primer**	Forward	Reverse
Flag-STAG2	CGGGATCCATGGATTACAAGGAT GACGACGATAAGATAGCAGCTCC AGAAATACC	CGGGATCCAAACATTGACACTC CAAGAAC
